# Combining Semi-Targeted Metabolomics and Machine Learning to Identify Metabolic Alterations in the Serum and Urine of Hospitalized Patients with COVID-19

**DOI:** 10.3390/biom13010163

**Published:** 2023-01-12

**Authors:** Gerard Baiges-Gaya, Simona Iftimie, Helena Castañé, Elisabet Rodríguez-Tomàs, Andrea Jiménez-Franco, Ana F. López-Azcona, Antoni Castro, Jordi Camps, Jorge Joven

**Affiliations:** 1 Unitat de Recerca Biomèdica, Hospital Universitari de Sant Joan, Institut d’Investigació Sanitària Pere Virgili, Universitat Rovira i Virgili, 43201 Reus, Spain; 2 Department of Internal Medicine, Hospital Universitari de Sant Joan, Institut d’Investigació Sanitària Pere Virgili, Universitat Rovira i Virgili, 43201 Reus, Spain

**Keywords:** biomarkers, COVID-19, machine learning, metabolomics, SARS-CoV-2

## Abstract

Viral infections cause metabolic dysregulation in the infected organism. The present study used metabolomics techniques and machine learning algorithms to retrospectively analyze the alterations of a broad panel of metabolites in the serum and urine of a cohort of 126 patients hospitalized with COVID-19. Results were compared with those of 50 healthy subjects and 45 COVID-19-negative patients but with bacterial infectious diseases. Metabolites were analyzed by gas chromatography coupled to quadrupole time-of-flight mass spectrometry. The main metabolites altered in the sera of COVID-19 patients were those of pentose glucuronate interconversion, ascorbate and fructose metabolism, nucleotide sugars, and nucleotide and amino acid metabolism. Alterations in serum maltose, mannonic acid, xylitol, or glyceric acid metabolites segregated positive patients from the control group with high diagnostic accuracy, while succinic acid segregated positive patients from those with other disparate infectious diseases. Increased lauric acid concentrations were associated with the severity of infection and death. Urine analyses could not discriminate between groups. Targeted metabolomics and machine learning algorithms facilitated the exploration of the metabolic alterations underlying COVID-19 infection, and to identify the potential biomarkers for the diagnosis and prognosis of the disease.

## 1. Introduction

Despite the lower pathogenicity of the Omicron variant, and the advances in vaccination in Western societies, the COVID-19 pandemic remains a global threat [1]. The total number of cases has risen from 300 million to more than 600 million worldwide between January and September 2022, and deaths have increased from approximately 5.5 million to 6.5 million. In addition, large sections of the population have not yet been vaccinated in low-income countries due to economic and logistical problems. Expert epidemiologists have the opinion that SARS-CoV-2 will continue to spread globally for many years to come [2]. Therefore, the pursuit of lines of research includes the mechanisms-of-action of SARS-CoV-2, the effects the infection has on the host’s metabolism, the search for biomarkers for the diagnosis and prognosis of infection, as well as the monitoring of disease evolution.

Viral infections cause major metabolic disturbances in the infected organism. Viruses need the host’s metabolic machinery for the synthesis of their own nucleic acids, proteins, lipids, and carbohydrates, and to obtain energy for viral replication [3]. In addition, they produce a strong viral immunological reaction, and may influence the gut host microbiome [4]. Metabolic dysregulation has been reported in patients infected with Zika, Dengue, Chikungunya, respiratory syncytial virus, SARS-CoV-1, and SARS-CoV-2 [5,6,7,8]. Conversely, the presence of chronic diseases of metabolic origin can influence viral infection. Indeed, patients with type II diabetes mellitus, cardiovascular disease, obesity, or cancer are at increased risk of developing severe COVID-19 [9,10]. Metabolomic studies are being widely used to seek a holistic understanding of pathological processes since it enables simultaneous analyses of hundreds, or thousands, of analytes in very limited volumes of the biological sample. The interpretation of the metabolic data generated through machine learning algorithms provides insight into the disease. The most relevant metabolic alterations are identified as are their interactions, as are the possible biomarkers and therapeutic targets [11]. Studies comparing the plasma metabolome of COVID-19-positive patients vs. healthy subjects have already been reported [12,13,14]. However, information on the specificity of the observed metabolic changes is scarce. For example, few studies have addressed the question of whether variations in circulating levels of the identified species are characteristic of COVID-19 infection or whether they may also be seen in other infectious, or inflammatory, diseases [15].

The present study used semi-directed metabolomics techniques and machine learning algorithms to analyze the concentrations of a broad panel of metabolites in the serum and urine of patients with COVID-19. The results were compared with those of healthy subjects, and patients with bacterial infectious diseases. Our aims were to evaluate the relationships between the alterations measured with the severity of the disease and comorbidities and to identify potential biomarkers.

## 2. Materials and Methods

### 2.1. Study Design and Participants

We conducted a post hoc retrospective cohort study in 126 patients hospitalized for COVID-19 between March and October 2020 in Hospital Universitari de Sant Joan. The inclusion criteria were: ≥18 years of age and to have a positive PCR result for COVID-19 obtained within 24 h before the samples for the study were drawn. The exclusion criteria were: having a life expectancy ≤ 24 h, impaired liver function, or pregnancy. We also tested samples from 45 COVID-19-negative patients hospitalized for bacterial infections. These samples, collected before the pandemic, belonged to a previous prospective study in patients with urinary catheter-related infections [16]. For the purposes of the present study, we selected a subgroup with an age and sex distribution to match, as closely as possible, the COVID-19-positive patients. As a control group, we analyzed samples from 50 healthy volunteers who had no clinical or biochemical evidence of diabetes, cancer, renal failure, liver disease, or neurological disorders [17]. A serum sample was obtained from all participants and a urine sample from COVID-19-positive and COVID-19-negative patients. Urine from healthy volunteers was not available. All samples were stored in our Biobank at −80 °C until the time of analyses. We recorded clinical and demographic data (Table S1) and calculated the McCabe score as an index of disease severity [18], and the Charlson index to categorize patients’ comorbidities [19]. This study was approved by the Comitè d’Etica i Investigació en Medicaments (Institutional Review Committee) of the Institut d’Investigació Sanitària Pere Virgili (CEIM Resolution 040/2018, modified on 16 April 2020).

### 2.2. Targeted Metabolomics

In all serum and urine samples, we measured the concentrations of molecules involved in the metabolism of amino acids, carbohydrates, cofactors, lipids, nucleotides, secondary metabolites, and xenobiotics. Briefly, samples were derivatized and analyzed on an Agilent 7890A gas chromatograph (Agilent Technologies, Santa Clara, CA, USA) coupled with an electron impact source to a 7200-quadrupole time-of-flight mass spectrometer equipped with a 7693 auto-sampler module and a J&W Scientific HP-5MS column (30 m × 0.25 mm, 0.25 µm; Agilent Technologies), as previously reported in detail [20]. The quantitative method developed was first validated in different human, animal, and cell conditions and in various biological samples (plasma, liver, adipose tissue, etc.). Therefore, the developed methodology accurately quantifies multiple metabolites involved in amino acids, carbohydrates, lipids, and nucleotide metabolism pathways. Data from serum and urine metabolites are expressed as µM and ISRR/mmol creatinine, respectively.

### 2.3. Statistical Analyses

The statistical significance of changes in metabolite concentrations was determined by the Wilcoxon rank-sum test followed by appropriate false-discovery rate (FDR q < 0.05) correction by the Benjamini-Hochberg method. All data from bar plots are shown as means and standard deviations. Some data are depicted with volcano plots and illustrate the representation of all measured metabolites, showing the mean log2 (fold change). A *p*-value below 0.05 was considered statistically significant. The Jupyter Notebook (version 6.0.1) was used to create volcano plots, while dimensionality reduction techniques developed machine learning classifier algorithms to stratify the study groups.

### 2.4. Dimensionality Reduction and Heatmap Analysis

Linear discriminant analysis (LDA) was used as a supervised method to reduce the dimension of the metabolomic dataset to focus on the different characteristics between groups. Moreover, heatmaps were employed to visualize the significant alterations between groups.

### 2.5. Machine Learning Analysis

Metabolomic structured and labeled datasets were analyzed with a gradient boost machine (GBM) classifier algorithm to find metabolites with the capacity to stratify among groups. Algorithm development and hyperparameter tuning were first evaluated with the training dataset (75% of the dataset). The hyperparameters “n_estimators [50, 100, 500, 1000]”, “max_features [‘auto’, ‘sqrt’, ‘log2′]”, “max_depth [None, 1, 3, 5, 10, 20]”, “subsample [0.5, 1]”, and “learning_rate [0.001, 0.01, 0.1]” were evaluated to find the optimal values for each argument and dataset (serum (COVID-19 vs. Control, COVID-19 vs. NoCOVID-19, NoCOVID-19 vs. Control), and urine (COVID-19 vs. NoCOVID-19)). Then, the model was automatically retrained with the best hyperparameters values and stored. Finally, the model was tested in the test dataset (25% of the dataset). 

Receiver operating characteristic (ROC) curves were employed to provide the performance of the classification model, and the quality of the model prediction was estimated by measuring the area under the ROC curve (AUROC). The Shapley Additive exPlanation (SHAP) method was used to identify and select the variables with the higher predictive values of each model. This method is a way of determining the contribution (termed SHAP value) of each variable to model outputs. The variables are classified according to their relative importance. We depicted the SHAP summary plots of the top 5 variables of the chosen prediction model. In plots, the further the value of a variable deviates from zero, the more impact it has on the model output. The scikit-learn package was used to develop tools for predictive data analysis and the SHAP package to figure out the results of the model. [21,22]. The used script is shown in Table S2.

## 3. Results

### 3.1. Comparisons between the Serum Metabolic Signatures of the Different Groups of Participants

**Figure 1:**
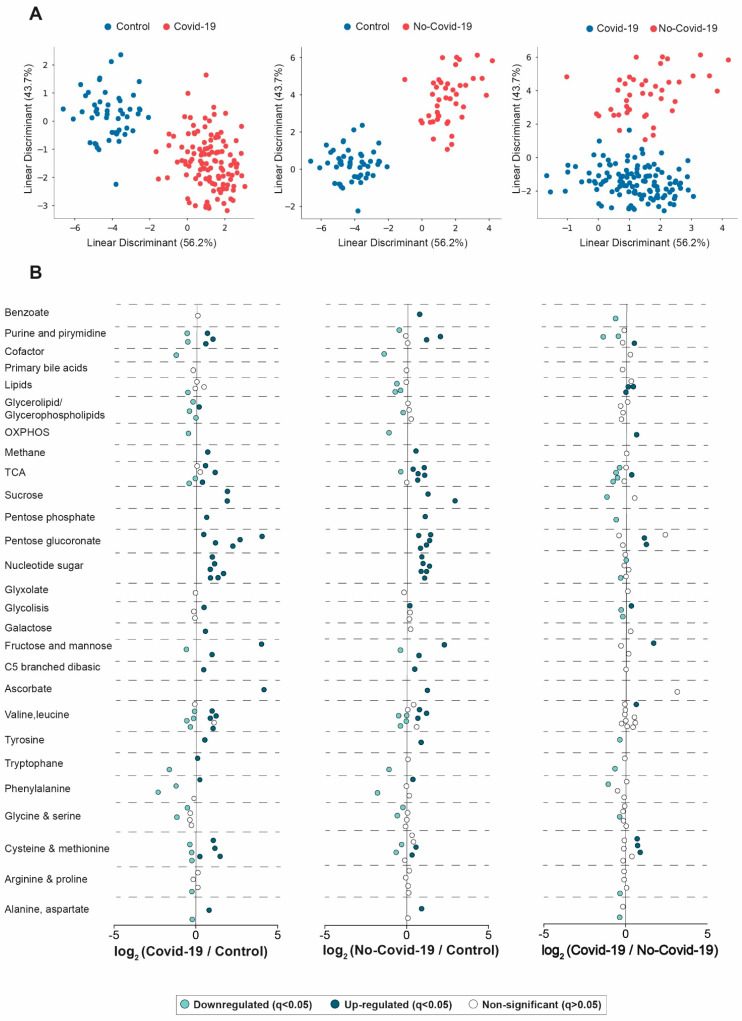
Serum metabolic signature in COVID-19-positive, negative patients and healthy volunteers. (**A**) Linear discriminant analysis showed a complete separation between groups. (**B**) Representation of the measured metabolites, showing the mean log2 (fold change). These graphs indicate, on the abscissa, the log2-fold change of the different metabolites between the two groups being compared. The magnitude of change and the *p*-value are taken into account to construct the graphs. For example, a positive log2-fold change of 2 indicates a 4-fold increase in a given variable in one group versus another. Serum data were transformed to molar percentage, and then the false-discovery rate (FDR q < 0.05) was calculated. Each metabolite was represented in sky-blue (significant decrease), blue (significant increase), and white (non-significant) dots. *p*-values < 0.05 were considered significant (Wilcoxon rank-sum test). OXPHOS: oxidative phosphorylation; TCA: tricarboxylic acid cycle.

The LDA was able to separate completely the metabolic signatures of COVID-19-positive and negative patients from the control group, and of COVID-19-positive patients from those with bacterial infections (Figure 1A). These results suggest that some metabolic alterations can be specific to COVID-19. Figure 1B and Tables S3–S5 show the magnitude of change in the different metabolic pathways. When patients (either positive or negative) were compared with the control group, we observed an increase in the concentrations of metabolites related to carbohydrate metabolism pathways, such as pentose phosphate, pentose and glucuronate interconversion, nucleotide sugars, and ascorbate and aldarate. The metabolism of amino acids was also altered, with increasing or decreasing metabolites involved in cysteine, methionine, alanine, aspartate, glycine, serine, phenylalanine, tryptophane, tyrosine, valine, and leucine biosynthesis. Conversely, when we compared COVID-19-positive patients against negative patients, we observed that positive patients had higher concentrations of metabolites involved in the pentose and glucuronate pathway and cysteine and methionine biosynthesis, while showing lower concentrations of metabolites involved in glycolysis, tricarboxylic acid cycle together with purine, pyrimidine, and phenylalanine biosynthesis.

### 3.2. Clinical Characteristics Associated with Changes in the Serum Metabolome

**Figure 2:**
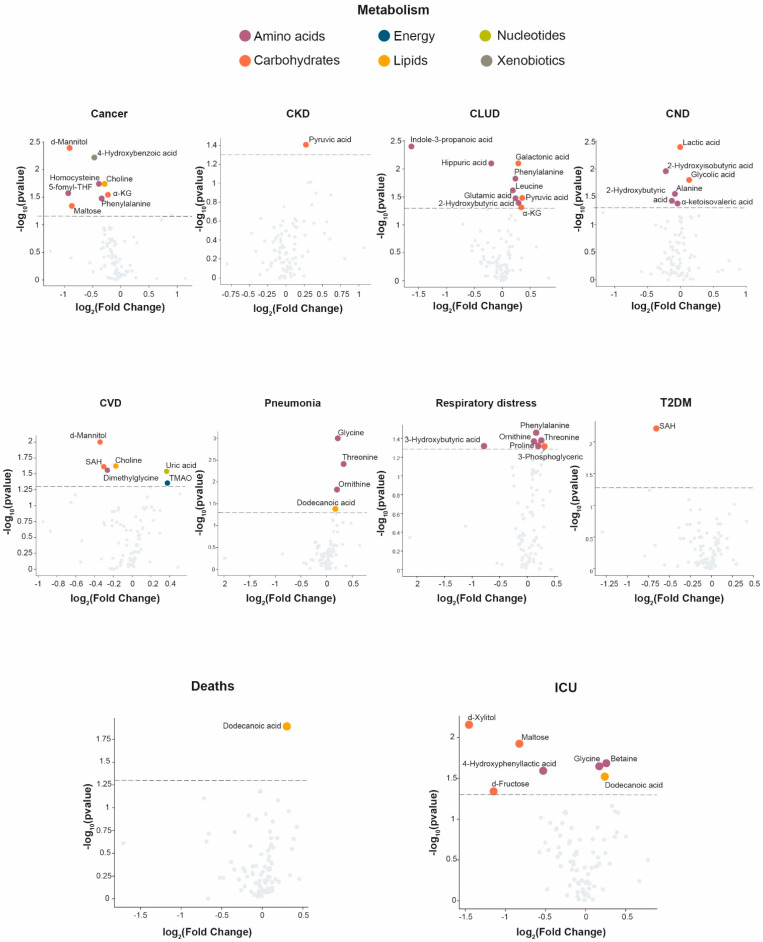
Influence of clinical characteristics on serum metabolome in COVID-19-positive patients. Volcano plots of clinical characteristics where significant metabolites were found are identified, and colored according to metabolite categories: Metabolism of amino acids, carbohydrates, energy, lipids, nucleotides, and xenobiotics. Serum data were transformed to molar percentage, and then the false-discovery rate (FDR q < 0.05) was calculated. *p* values < 0.05 were considered significant (Wilcoxon-rank sum test). CKD: chronic kidney disease; CLUD: chronic lung disease; CND: chronic neurological disease; CVD: cardiovascular disease; T2DM: type 2 diabetes mellitus; ICU: intensive care unit.

We studied the impact of comorbidities and factors related to disease severity on the concentrations of metabolites in patients with COVID-19 (Figure 2). Patients with cancer had lower levels of molecules associated with carbohydrate, amino acid, and xenobiotic metabolism. Patients with type 2 diabetes mellitus showed lower levels of S-adenosylhomocysteine. Moreover, we found that patients with chronic lung disease, neurological diseases, and respiratory distress showed higher levels of metabolites involved in the metabolism of amino acids and carbohydrates. Patients with cardiovascular disease showed lower levels of metabolites associated with the metabolism of amino acids, carbohydrates, and lipids, and increased metabolites associated with the metabolism of nucleotides and energy metabolism. Finally, we observed that patients admitted to the Intensive Care Unit showed decreased levels of metabolites related to carbohydrate metabolisms, such as d-xylitol, maltose, and fructose, and increased levels of metabolites involved in amino acid and lipid metabolisms, such as glycine, betaine, and dodecanoic (lauric) acid. Patients who died had higher concentrations of lauric acid than surviving patients.

### 3.3. Comparisons between the Urine Metabolic Signatures of COVID-19-Positive and Negative Patients

**Figure 3:**
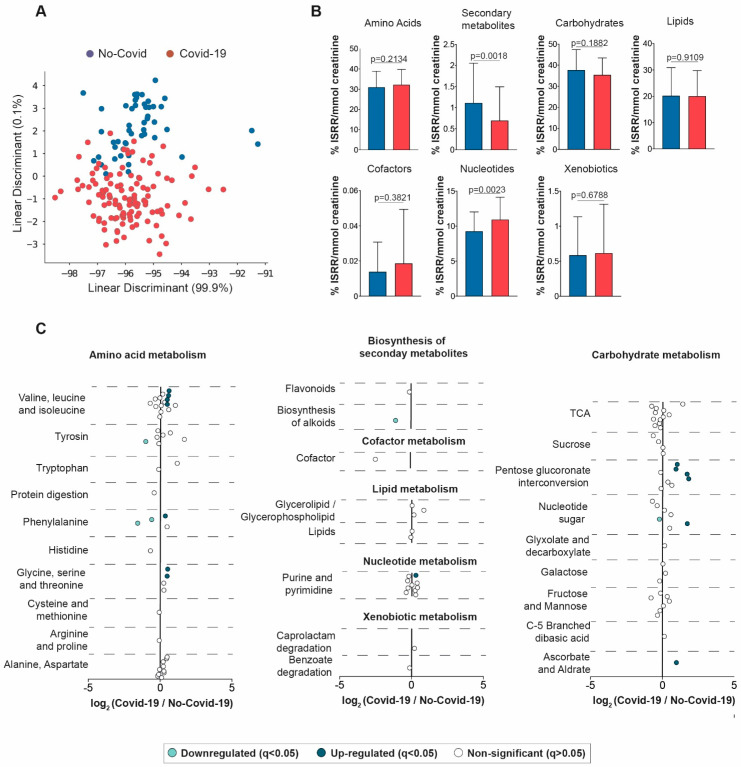
Urinary metabolic signature in COVID-19-positive and negative patients. (**A**) Linear discriminant analysis showed a considerable degree of overlapping between groups. (**B**) Variations in the metabolite group levels in COVID-19-positive and negative patients. (**C**) Representation of all measured metabolites, showing the mean log2 (fold change). These graphs indicate on the abscissa axis the log2-fold change of the different metabolites between the two groups being are compared. The magnitude of change and the *p*-value are taken into account to construct the graphs. For example, a positive log2-fold change of 2 indicates a 4-fold increase in a given variable in one group versus another. Urine data were transformed to molar percentage, and then the false-discovery rate (FDR q < 0.05) was calculated. Each metabolite was represented in sky-blue (significant decrease), blue (significant increase), and white (non-significant) dots. *p*-values < 0.05 were considered significant (Wilcoxon rank-sum test). Bar plots represent means and standard deviations. TCA: tricarboxylic acid cycle.

LDA showed a high degree of overlap in the metabolic signatures of both groups (Figure 3A). The main alterations were produced in the biosynthesis of secondary metabolites and nucleotide metabolism, which decreased and increased, respectively, in positive patients compared to the negative patients (Figure 3B). Other alterations included pentose glucuronate interconversion, nucleotide sugars, ascorbate, and several amino acids (Figure 3C and Table S6). Overall, the changes observed in urine reflected, to some extent, those found in serum, but the differences were much smaller and did not allow for segregation between groups.

### 3.4. Machine Learning Potential Identified in COVID-19 Biomarkers in Serum, but Not in Urine

**Figure 4:**
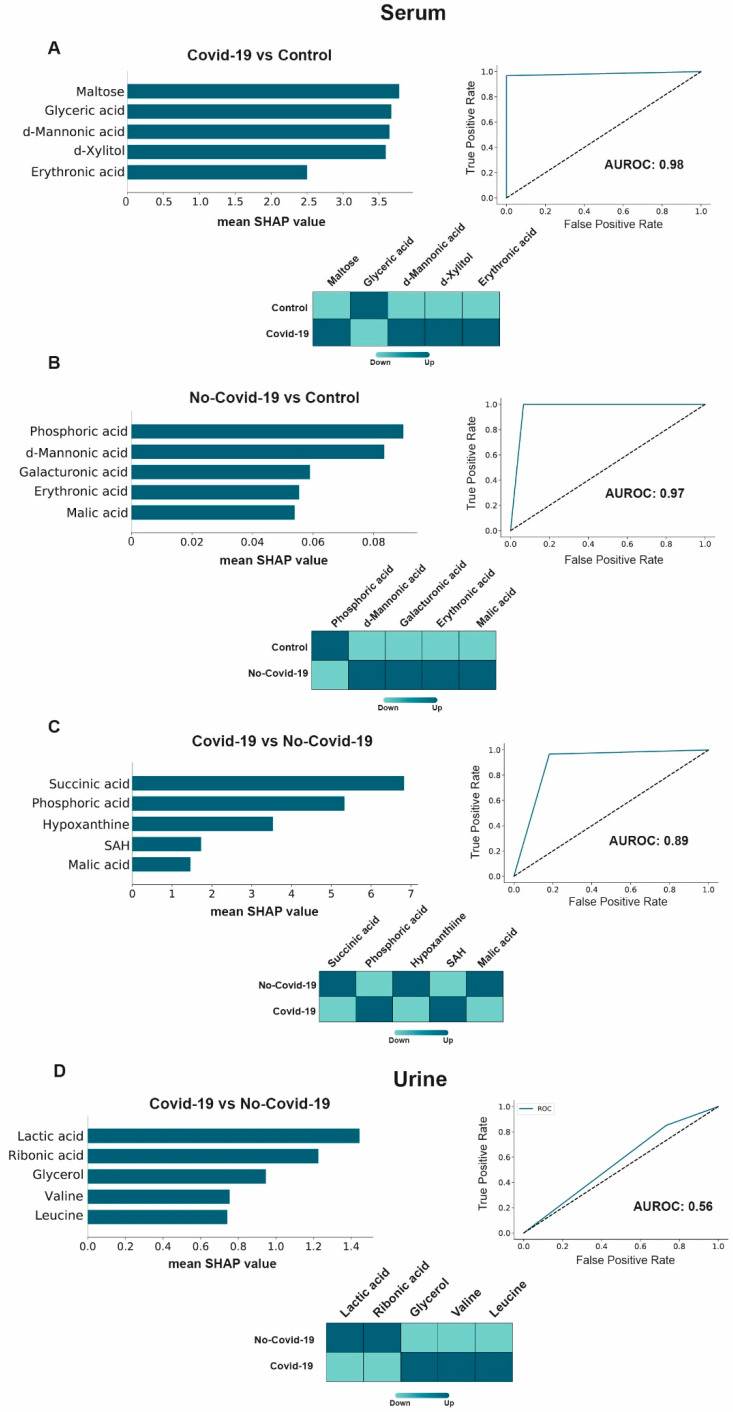
Machine learning shows the utility of serum, but not urine, in the diagnosis of COVID-19. SHapley Additive exPlanations (SHAP). Summary plots of the gradient boosting machine shows the top 5 metabolites discriminating between the different categories (left panels). The model accuracy was estimated by receiver operating characteristics plots, and the areas under the curve (AUROC) were calculated (right panels). The type of variation (increase or decrease) is shown in heatmaps (bottom panels). (**A**) Serum metabolites, COVID-19-positive patients vs. control group. (**B**) Serum metabolites, COVID-19-negative patients vs. control group. (**C**) Serum metabolites, COVID-19-positive vs. negative patients. (**D**) Urinary metabolites, COVID-19-positive vs. negative patients.

The GBM algorithm identified maltose, glyceric acid, mannonic acid, xylitol, and erythronic acid as the metabolites with the highest capacity to discriminate COVID-19-positive patients from the control group (Figure 4A). These parameters were increased in positive patients except for glyceric acid, which was decreased. In contrast, when we compared COVID-19-negative patients with the control group, we found that the top five metabolites were phosphoric, mannonic, galacturonic, erythronic, and malic acids, all of which increased in COVID-19-negative patients, except for phosphoric acid, which decreased (Figure 4B). The algorithm was also employed to identify metabolites able to discriminate between COVID-19-positive and negative patients, and found that serum succinate had high diagnostic accuracy and ability to segregate both groups (Figure 4C). This parameter was decreased in COVID-19 patients. In contrast, none of the urinary parameters was able to distinguish between positive and negative patients (Figure 4D).

## 4. Discussion

**Figure 5:**
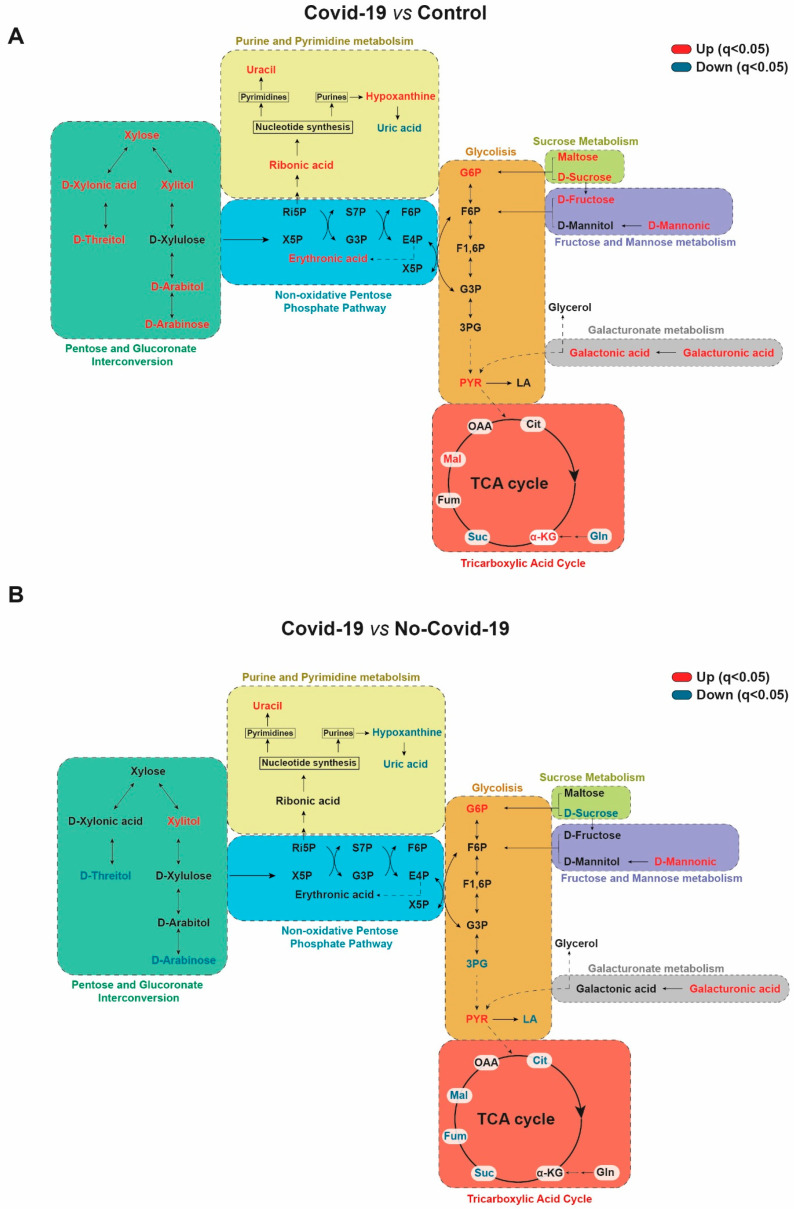
Simplified scheme showing the alterations in the metabolic pathways studied when COVID-19-positive patients are compared with the control group (**A**) and with COVID-19-negative patients (**B**). Red color highlights uncreased metabolites, while blue color shows decreased metabolites. 3PG: 3-phosphoglycerate; α-KG: α-Ketoglutarate; Cit: Citrate; E-4-P: Erythrose-4-phosphate; F1,6P: Fructose-1,6-biphosphate; F6P: Fructose-6-phosphate; Fum: Fumarate; G3P: Gln: Glutamine; Glyceraldehyde-3-phosphate; LA: Lactate; Mal: Malate; OAA: Oxaloacetate; PYR: Pyruvate; Ri-5-P: Ribulose-5-phosphate; S-7-P: Sedoheptulose-7-phosphate; Suc: Succinate; X-5-P: Xylulose-5-phosphate.

We observed striking differences between the metabolic signatures of healthy subjects, COVID-19-positive patients, and COVID-19-negative patients. The main metabolic pathways altered in the sera of COVID-19 patients were pentose glucuronate interconversion, ascorbate and fructose metabolism, nucleotide sugar pathway, as well as nucleotide and amino acid metabolism. Further machine learning identified several individual parameters that distinguished positive from negative COVID-19 patients, and control subjects. These results suggest a profound alteration of pathways related to energy metabolism, and the synthesis of nucleotides and amino acids. These pathways are closely related and have numerous interactions between them (Figure 5).

Overall, our results suggest an activation of the glycolytic cascade and an increase in glucose-6-phosphate concentrations; a metabolite that serves as a branching point between glycolysis, pentose phosphate pathway, pentose and glucuronate interconversion [23]. Viral infections redirect the metabolism of host cells to promote the synthesis of new viral particles. One of the key enzymes of viral replication is RNA-dependent RNA polymerase, which acts through the nucleotide addition cycle composed of multiple functional states involving conformational changes of both protein and nucleotides [24]. Viral transcription obtains energy and substrates for the synthesis of structural particles from boosting aerobic glycolysis and the pentose phosphate pathway [25]. The stimulation of aerobic glycolysis leads to an increase in the activity of hexokinase, the rate-limiting enzyme of glycolysis, and favoring the stimulation of the pentose phosphate pathway. The role of hexokinase is to convert glucose into glucose-6-phosphate, which is subsequently oxidized by glucose-6-phosphate dehydrogenase (G6PD) in the pentose phosphate pathway to synthesize ribose-5-phosphate, required for nucleic acid synthesis, sugar phosphate precursors that are necessary for the synthesis of amino acids, and NADPH (Figure 5) [26,27]. Many viruses, including the influenza virus, hepatitis C virus, and HIV-1, can upregulate the pentose phosphate pathway [28,29], and our results agree with the recent suggestion that SARS-CoV-2 may do the same [30].

Of all these metabolic pathways, the most clearly representative of COVID-19-positive patients is that of pentose and glucuronate interconversion, which shows a great increase relative to negative patients, and the control group. This is a detoxification pathway in which d-glucuronic acid binds to hydroxyl or the amino groups of toxic substances under the catalysis of UDP-glucuronosyltransferase to increase water solubility and allow their release within bile, or urine [31]. Our results are novel in that very little has been published on the alterations in this pathway in COVID-19 patients. However, recent studies have linked an increase in the pentose glucuronate interconversion with alterations in the microbiome of patients with mouth infections [32,33]. Further, pharmacological studies have reported that the effects of some anti-inflammatory agents are mediated through the modulation of this metabolic pathway in humans and experimental animals [34,35].

We sought to assess if there were differences in metabolite levels in COVID-19 patients based on their comorbidities and their severity. In our opinion, the most relevant results were those referring to the severity of the disease. Volcano plots showed that patients who required intensive care and those who died had had higher serum concentrations of lauric acid. Ingested lauric acid from oils is transformed by the human body into laurate-monoglyceride that inactivates lipid-coated viruses by binding to the viral envelope, thereby preventing the attachment and entry into the host cells [36,37]. Evidence has also been reported suggesting that this compound disintegrates the viral envelope and kills the virus [38]. Our results, therefore, may seem counterintuitive, since we have observed that the most severe patients had higher concentrations of lauric acid. One possible explanation is that this compound does not exert this virucidal function when it is not bound to glycerol. In this case, higher concentrations of free lauric acid could be associated with lower concentrations of laurate-monoglyceride. Another explanation is that the levels of lauric acid are increased to synthesize more laurate monoglyceride in order to try to counteract the viral infection. In any case, this opens an interesting line of research on the relationships between lauric acid and the severity of COVID-19. In addition, patients who needed intensive care had lower xylitol concentrations than those who did not. Xylitol is a product of pentose and glucoronate interconversion pathway and has inflammatory, antiglycemic, antiviral, and antibacterial properties in lung infections [39]. Xylitol has been reported to decrease the concentration of salts in the airway surface liquid lining the interior of the lungs, improving antibody activity [40]. An in vitro study showed that this compound has anti-inflammatory properties. Xylitol-treated macrophages had 10 times less adhesion capacity than control subjects and lower levels of cell adhesion molecules; important because cell adhesion is a crucial step in the pulmonary inflammatory response [41]. Reports have highlighted that the dietetic administration of xylitol reduces the viral load in mice infected with the human respiratory syncytial virus [40] or the influenza A virus infection [42].

In our study, the application of artificial intelligence algorithms helped distinguish the individual metabolites that have the greatest ability to discriminate between the different study groups and help identify potential biomarkers. An increase in maltose concentrations was the alteration with the best ability to discriminate between patients with COVID-19 and the control group. A decrease in succinate was the metabolite with the best ability to discriminate between positive and negative COVID-19 patients. Xylitol, glyceric, mannonic, and erythronic acids had similar power of discrimination as maltose. These results are not easy to explain. Maltose, mannonic, and erythronic acids are products of plant metabolism and, although they are ingested in the diet, they are not synthesized in relevant amounts by the human body. Perhaps the explanation of why these metabolites have altered concentrations is related to the effects of infection on the gut microbiota. Indeed, the existence of a gut-lung axis has been postulated, with implications in human pathology that are reflected in changes in the circulating concentrations of metabolites [43]. Dysbiosis in gut microbiota is associated with lung disorders and respiratory infections [44]. The depletion of certain bacteria within the gut microbiota due to antibiotic intake influences lung diseases [45] and conversely, changes in lung microbes influence the composition of gut microbiota [46]. Several studies reported that changes in the serum levels of maltose, mannose, succinic acid, and erythronic acid are associated with changes in gastrointestinal microbiome [47,48,49,50,51]. Moreover, a recent multiomics study showed multiple gut microbe–metabolite–cytokine interrelationships in COVID-19 [52]. It is worth noting that changes in the microbiome have been associated with alterations in the levels of pentose glucuronate interconversion metabolites [53,54,55].

Metabolomics studies play an important role in the investigation of the molecular bases of non-communicable and infectious diseases and have revealed themselves as a powerful tool in the study of COVID-19 to better understand the mode of action of the virus and achieve more accurate treatments [56]. These methods have been used to investigate the differences between mild and moderate COVID-19 patients, suggesting that a moderate disease state may provide the most effective setting for therapeutic intervention [57]. In addition, studies by our research group [11] and by other authors [58] have used targeted and untargeted metabolomics to analyze the plasma lipidome and metabolome in COVID-19 patients and healthy controls, identifying molecules related to infection and disease severity. On the other hand, metabolomics has been used to successfully construct diagnostic models that predict COVID-19 infection risk and disease severity [59]. Finally, integrating metabolomics into multiomics analyses has been able to provide a landscape for COVID-19 patients without comorbidities [60].

## 5. Conclusions

Alterations in serum maltose, mannonic acid, xylitol, or glyceric acid segregate positive patients from the control group with high diagnostic accuracy, while succinic acid segregates positive patients from those with infectious diseases of another origin. These parameters, therefore, could be good markers for the diagnosis of COVID-19. Conversely, an increase in the concentration of lauric acid could be a marker for the prognosis of the disease. Since urine samples are relatively easy to obtain, laboratory measurements could be made in order to identify urinary biomarkers. However, although the changes in serum concentrations of metabolites are reflected in the urine, they are small and do not provide us with effective indices for the nuanced evaluation of the disease. Semi-targeted metabolomics interpreted using machine learning algorithms has allowed us to delve into the metabolic alterations underlying COVID-19 and identify potential biomarkers for its diagnosis and prognosis.

## Data Availability

The data presented in this study are available from the corresponding author upon reasonable request.

## Supplementary Materials

The following supporting information can be downloaded at: https://www.mdpi.com/article/10.3390/biom13010163/s1, Table S1: Demographic and clinical characteristics of the patients and the healthy subjects; Table S2: Gradient Boosting Machine scripts; Table S3: Serum metabolites from healthy volunteers and COVID-19 positive patients; Table S4: Serum metabolites from healthy volunteers and COVID-19 negative patients; Table S5: Serum metabolites from COVID-19 positive and negative patients; Table S6: Urine metabolites from COVID-19 positive and negative patients.
