# Supplementary material for: Combining Semi-Targeted Metabolomics and Machine Learning to Identify Metabolic Alterations in the Serum and Urine of Hospitalized Patients with COVID-19
Source: Biomolecules. 2023 Jan 12;13(1):163. doi: 10.3390/biom13010163 (PMC9856035; doi:10.3390/biom13010163)
Supplement: Supplementary file 1 [file biomolecules-13-00163-s001.zip › biomolecules-2086203-supplementary.pdf]

Supplementary Table S1. Demographic and clinical characteristics of the patients and the healthy subjects

	Healthy subjects (n = 50)	COVID-19 negative patients (n = 45)	COVID-19 positive patients (n = 126)
**Demographic variables**			
Sex, male	38 (76.0)	30 (66.7)	68 (54.8) [a]
Age, years	75 (66- 84)	84 (75- 89) [b]	71 (58-83)
Smoking, n (%)	19 (38.0)	16 (35.6)	6 (4.8) [b,d]
Alcohol intake, n (%)	28 (56.0)	7 (15.5) [b]	6 (4.8) [b,c]
**Comorbidities**			
Type 2 diabetes mellitus, n (%)	0	22 (48.9)	30 (23.8) [e]
Cardiovascular disease, n (%)	0	18 (40)	68 (54)
Chronic liver disease, n (%)	0	0	1 (0.8)
Chronic lung disease, n (%)	0	0	18 (14.3)
Chronic kidney disease, n (%)	0	19 (42.2)	22 (17.5) [d]
Chronic neurological disease n (%),	0	0	29 (23)
Cancer, n (%)	0	17 (37.8)	16 (12.7) [e]
**Charlson index**			
No comorbidity, n (%)	NA	10 (22.2)	83 (65.9) [e,*]
Low comorbidity, n (%)		18 (40.0)	29 (23.0)
High comorbidity, n (%)		17 (37.8)	14 (11.1)
**McCabe index**			
RFD, n (%)	NA	10 (22.2)	7 (5.6) [e, *]
UFD, n (%)		19 (42.2)	31 (24.6)
NFD, n (%)		16 (35.6)	88 (69.8)
**Medications**			
ACEIs, n (%)	NA	14 (31.1)	24 (27.0)
ARAs, n (%)	NA	12 (26.7)	21 (16.7)
Oral antidiabetics, n (%)	NA	19 (42.2)	37 (29.4)
Insulin, n (%)	NA	9 (20.0)	28 (22.2)
Statins, n (%)	NA	16 (35.6)	44 (34.9)

[a] P < 0.01, [b] P < 0.001, with respect to healthy subjects; [c] P < 0.05, [d] P < 0.01, [e] P < 0.001 with respect to COVID-19 negative patients. *: Global P-value including the three categories of each index. Statistical analyses performed by the Student's *t* test (quantitative) or the χ-square test (qualitative). Results are given as medians and 95% CI or as numbers and percentages. ACEIs: Angiotensin converting enzyme inhibitors; ARAs, Angiotensin II receptor antagonists; NFD: Non-fatal disease; RFD: Rapidly fatal disease. UFD: Ultimately fatal disease

Supplementary Table S2. Gradient Boosting Machine scripts

See next pages.

In [1]:
```python
# Data treatment
# ==============================================================================
import numpy as np
import pandas as pd
import statsmodels.api as sm

# Graphs
# ==============================================================================
import matplotlib.pyplot as plt

# Processing and modeling
# ==============================================================================
from sklearn.datasets import load_boston
from sklearn.ensemble import GradientBoostingClassifier
from sklearn.metrics import accuracy_score
from sklearn.metrics import confusion_matrix
from sklearn.compose import ColumnTransformer
from sklearn.preprocessing import OneHotEncoder
from sklearn.model_selection import cross_val_score
from sklearn.model_selection import train_test_split
from sklearn.model_selection import RepeatedKFold
from sklearn.model_selection import GridSearchCV
from sklearn.model_selection import ParameterGrid
from sklearn.inspection import permutation_importance
import multiprocessing

# Warnings configuration
# ==============================================================================
import warnings
warnings.filterwarnings('once')
```

In [5]:
```python
datos = pd.read_csv("Cov_Contr_Pl.csv")
```

```
/Users/gerard/opt/anaconda3/lib/python3.8/site-packages/ipykernel/ipkernel.py:287: DeprecationWarning: `should_run_async` will not call `transform_cell` automatically in the future. Please pass the result to `transformed_cell` argument and any exception that happen during thetransform in `preprocessing_exc_tuple` in IPython 7.17 and above.
  and should_run_async(code)
```

In [6]:
```python
# Division of data into train and test
# ==============================================================================
X_train, X_test, y_train, y_test = train_test_split(
                                        datos.drop(columns = 'Group'),
                                        datos['Group'],
                                        random_state = 123
                                    )

# One-hot-encoding of categorical variables
# ==============================================================================
# The name of the numerical and categorical columns is identified
cat_cols = X_train.select_dtypes(include=['object', 'category']).columns.to_list()
numeric_cols = X_train.select_dtypes(include=['float64', 'int']).columns.to_list()

# One-hot-encoding is applied only to categorical columns
preprocessor = ColumnTransformer(
                    [('onehot', OneHotEncoder(handle_unknown='ignore'), cat_cols)],
                    remainder='passthrough'
                )

# Once the ColumnTransformer object has been defined, with the fit() method
# method, the transformations are learned with the training data and are applied to
# the two sets with transform(). Both operations at the same time with fit_transform().
X_train_prep = preprocessor.fit_transform(X_train)
X_test_prep  = preprocessor.transform(X_test)
```

In [7]:
```python
# Grid of evaluated hyperparameters
# ==============================================================================
param_grid = {'n_estimators'  : [50, 100, 500, 1000],
              'max_features'  : ['auto', 'sqrt', 'log2'],
              'max_depth'     : [None, 1, 3, 5, 10, 20],
              'subsample'     : [0.5, 1],
              'learning_rate' : [0.001, 0.01, 0.1]
             }

# Grid search with cross validation
# ==============================================================================
grid = GridSearchCV(
        estimator  = GradientBoostingClassifier(random_state=123),
        param_grid = param_grid,
        scoring    = 'accuracy',
        n_jobs     = multiprocessing.cpu_count() - 1,
        cv         = RepeatedKFold(n_splits=3, n_repeats=1, random_state=123),
        refit      = True,
        verbose    = 0,
        return_train_score = True
       )

grid.fit(X = X_train_prep, y = y_train)

# Results
# ==============================================================================
resultados = pd.DataFrame(grid.cv_results_)
resultados.filter(regex = '(param*|mean_t|std_t)') \
    .drop(columns = 'params') \
    .sort_values('mean_test_score', ascending = False) \
    .head(4)
```

Out[7]:

	param_learning_rate	param_max_depth	param_max_features	param_n_estimators	param_subsample	mean_test_score	std_test_score	mean_train_score	std_train_score
**429**	0.1	20	log2	500	1	0.976375	0.000261	1.0	0.0
**309**	0.1	None	log2	500	1	0.976375	0.000261	1.0	0.0
**405**	0.1	10	log2	500	1	0.976375	0.000261	1.0	0.0
**207**	0.01	3	sqrt	1000	1	0.976190	0.019440	1.0	0.0

In [8]:
```python
# Better hyperparameters by cross-validation
# ==============================================================================
print("----------------------------------------")
print("Mejores hiperparámetros encontrados (cv)")
print("----------------------------------------")
print(grid.best_params_, ":", grid.best_score_, grid.scoring)
```

```
----------------------------------------
Mejores hiperparámetros encontrados (cv)
----------------------------------------
{'learning_rate': 0.1, 'max_depth': None, 'max_features': 'log2', 'n_estimators': 500, 'subsample': 1} : 0.976375046142488 accuracy
/Users/gerard/opt/anaconda3/lib/python3.8/site-packages/ipykernel/ipkernel.py:287: DeprecationWarning: `should_run_async` will not call `transform_cell` automatically in the future. Please pass the result to `transformed_cell` argument and any exception that happen during thetransform in `preprocessing_exc_tuple` in IPython 7.17 and above.
  and should_run_async(code)
```

In [9]:
```python
modelo_final = grid.best_estimator_
```

In [10]:
```python
# Final model test error
# ==============================================================================
predicciones = modelo_final.predict(X = X_test_prep)
predicciones[:10]
```

Out[10]:
```
array([1, 1, 1, 1, 1, 1, 1, 1, 1, 1])
```

In [11]:
```python
mat_confusion = confusion_matrix(
                    y_true    = y_test,
                    y_pred    = predicciones
                )

accuracy = accuracy_score(
            y_true    = y_test,
            y_pred    = predicciones,
            normalize = True
           )

print("Matriz de confusión")
print("-------------------")
print(mat_confusion)
print("")
print(f"El accuracy de test es: {100 * accuracy} %")
```

```
Matriz de confusión
-------------------
[[12  0]
 [ 1 30]]

El accuracy de test es: 97.67441860465115 %
/Users/gerard/opt/anaconda3/lib/python3.8/site-packages/ipykernel/ipkernel.py:287: DeprecationWarning: `should_run_async` will not call `transform_cell` automatically in the future. Please pass the result to `transformed_cell` argument and any exception that happen during thetransform in `preprocessing_exc_tuple` in IPython 7.17 and above.
  and should_run_async(code)
```

In [42]:
```python
import seaborn as sns
import matplotlib.pyplot as plt

ax= plt.subplot()
sns.heatmap(mat_confusion, annot=True, ax = ax, cmap=plt.cm.Reds); #annot=True to annotate cells

# labels, title and ticks
ax.set_xlabel('Predicted labels');ax.set_ylabel('True labels');
ax.set_title('Confusion Matrix');
ax.xaxis.set_ticklabels(['Control', 'Covid']); ax.yaxis.set_ticklabels(['Control', 'Covid']);
plt.savefig('Matrix_Pl_Cov_Cont.pdf', format= 'pdf', dpi=300, transparent = True, bbox_inches='tight')
```

```
`should_run_async` will not call `transform_cell` automatically in the future. Please pass the result to `transformed_cell` argument and any exception that happen during thetransform in `preprocessing_exc_tuple` in IPython 7.17 and above.
```

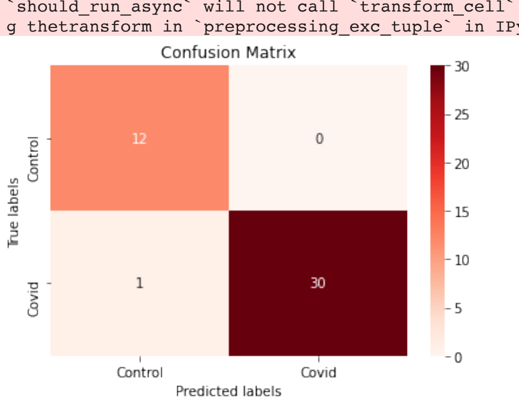

In [28]:
```python
# roc curve and auc score
from sklearn.datasets import make_classification
from sklearn.neighbors import KNeighborsClassifier
from sklearn.ensemble import RandomForestClassifier
from sklearn.model_selection import train_test_split
from sklearn.metrics import roc_curve
from sklearn.metrics import roc_auc_score

def plot_roc_curve(fpr, tpr):
    plt.plot(fpr, tpr, color='gold', label='ROC')
    plt.plot([0, 1], [0, 1], color='darkblue', linestyle='--')
    plt.xlabel('False Positive Rate')
    plt.ylabel('True Positive Rate')
    plt.title('Receiver Operating Characteristic (ROC) Curve')
    plt.legend()
    plt.savefig('AUC_Pl_Cov_Cont.pdf', format= 'pdf', dpi=300, transparent = True, bbox_inches='tight')
```

```
`should_run_async` will not call `transform_cell` automatically in the future. Please pass the result to `transformed_cell` argument and any exception that happen during thetransform in `preprocessing_exc_tuple` in IPython 7.17 and above.
```

In [29]:
```python
probs = modelo_final.predict(X_test_prep)
```

In [30]:
```python
auc = roc_auc_score(y_test, probs)
print('AUC: %.2f' % auc)
```

```
AUC: 0.98
`should_run_async` will not call `transform_cell` automatically in the future. Please pass the result to `transformed_cell` argument and any exception that happen during thetransform in `preprocessing_exc_tuple` in IPython 7.17 and above.
```

In [31]:
```python
fpr, tpr, thresholds = roc_curve(y_test, probs)

plot_roc_curve(fpr,tpr)
```

```
`should_run_async` will not call `transform_cell` automatically in the future. Please pass the result to `transformed_cell` argument and any exception that happen during thetransform in `preprocessing_exc_tuple` in IPython 7.17 and above.
```

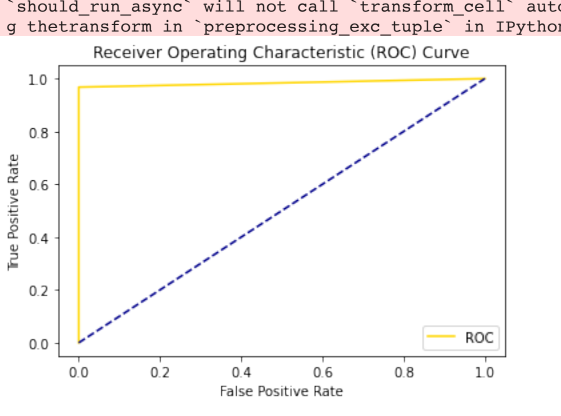

In [17]:
```python
# Probability prediction
# ==============================================================================
predicciones = modelo_final.predict_proba(X = X_test_prep)
predicciones[:5, :]
```

```
/Users/gerard/opt/anaconda3/lib/python3.8/site-packages/ipykernel/ipkernel.py:287: DeprecationWarning: `should_run_async` will not call `transform_cell` automatically in the future. Please pass the result to `transformed_cell` argument and any exception that happen during thetransform in `preprocessing_exc_tuple` in IPython 7.17 and above.
  and should_run_async(code)
```

Out[17]:
```
array([[9.95403759e-12, 1.00000000e+00],
       [1.33226763e-15, 1.00000000e+00],
       [3.33066907e-15, 1.00000000e+00],
       [2.66453526e-15, 1.00000000e+00],
       [1.15945744e-05, 9.99988405e-01]])
```

In [18]:
```python
# Classification using the highest probability class
# ==============================================================================
df_predicciones = pd.DataFrame(data=predicciones, columns=['0', '1'])
df_predicciones['clasificacion_default_0.5'] = np.where(df_predicciones['0'] > df_predicciones['1'], 0, 1)
df_predicciones.head(3)
```

```
/Users/gerard/opt/anaconda3/lib/python3.8/site-packages/ipykernel/ipkernel.py:287: DeprecationWarning: `should_run_async` will not call `transform_cell` automatically in the future. Please pass the result to `transformed_cell` argument and any exception that happen during thetransform in `preprocessing_exc_tuple` in IPython 7.17 and above.
  and should_run_async(code)
```

Out[18]:

	0	1	clasificacion_default_0.5
**0**	9.954038e-12	1.0	1
**1**	1.332268e-15	1.0	1
**2**	3.330669e-15	1.0	1

In [19]:
```python
# Final classification using a threshold of 0.8 for class 1.
# ==============================================================================
df_predicciones['clasificacion_custom_0.8'] = np.where(df_predicciones['1'] > 0.9, 1, 0)
df_predicciones[df_predicciones['clasificacion_custom_0.8']!=df_predicciones['clasificacion_default_0.5']] \
    .head(3)
```

```
/Users/gerard/opt/anaconda3/lib/python3.8/site-packages/ipykernel/ipkernel.py:287: DeprecationWarning: `should_run_async` will not call `transform_cell` automatically in the future. Please pass the result to `transformed_cell` argument and any exception that happen during thetransform in `preprocessing_exc_tuple` in IPython 7.17 and above.
  and should_run_async(code)
```

Out[19]:

0	1	clasificacion_default_0.5	clasificacion_custom_0.8

In [20]:
```python
importancia_predictores = pd.DataFrame(
                            {'predictor': datos.drop(columns = "Group").columns,
                             'importancia': modelo_final.feature_importances_}
                            )
print("Importancia de los predictores en el modelo")
print("-------------------------------------------")
importancia_predictores.sort_values('importancia', ascending=False)
```

```
Importancia de los predictores en el modelo
-------------------------------------------
/Users/gerard/opt/anaconda3/lib/python3.8/site-packages/ipykernel/ipkernel.py:287: DeprecationWarning: `should_run_async` will not call `transform_cell` automatically in the future. Please pass the result to `transformed_cell` argument and any exception that happen during thetransform in `preprocessing_exc_tuple` in IPython 7.17 and above.
  and should_run_async(code)
```

Out[20]:

	predictor	importancia
**34**	Erythronic acid	1.789223e-01
**22**	Glyceric acid	9.690568e-02
**44**	d-Xylitol	9.163407e-02
**62**	Galacturonic acid	8.760900e-02
**30**	d-Threitol	7.278913e-02
**...**	...	...
**79**	Choline	4.409360e-07
**1**	Lactic acid	4.235612e-07
**14**	Leucine	3.247883e-07
**52**	3-Phosphoglyceric acid	1.493937e-09
**71**	Oleic acid	2.037395e-11

85 rows × 2 columns

In [23]:
```python
import shap
shap.initjs()
explainer = shap.TreeExplainer(modelo_final)
```

In [27]:
```python
import numpy as np
import matplotlib as mpl
import matplotlib.pyplot as plt
from matplotlib import cm

from collections import OrderedDict

shap.summary_plot(shap_values, X_test,plot_type="bar", max_display= 5, show= False)
plt.savefig('SHAP_Pl_Cov_Cont.pdf', format= 'pdf', dpi=300, transparent = True, bbox_inches='tight')
```

```
`should_run_async` will not call `transform_cell` automatically in the future. Please pass the result to `transformed_cell` argument and any exception that happen during thetransform in `preprocessing_exc_tuple` in IPython 7.17 and above.
```

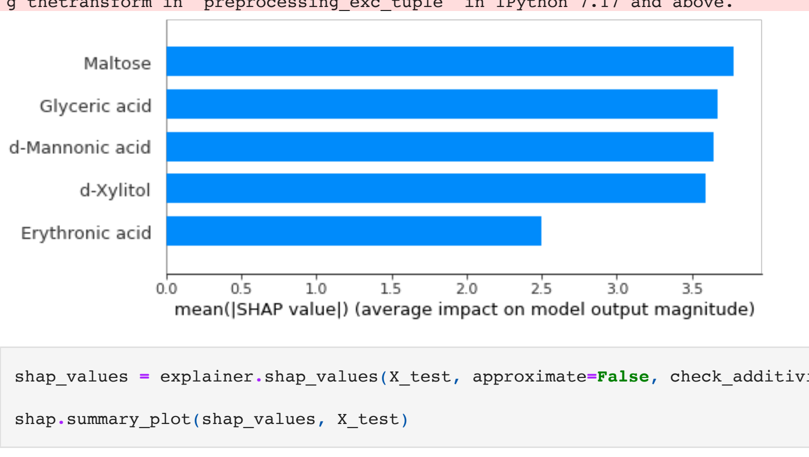

In [44]:
```python
shap_values = explainer.shap_values(X_test, approximate=False, check_additivity=False)

shap.summary_plot(shap_values, X_test)
```

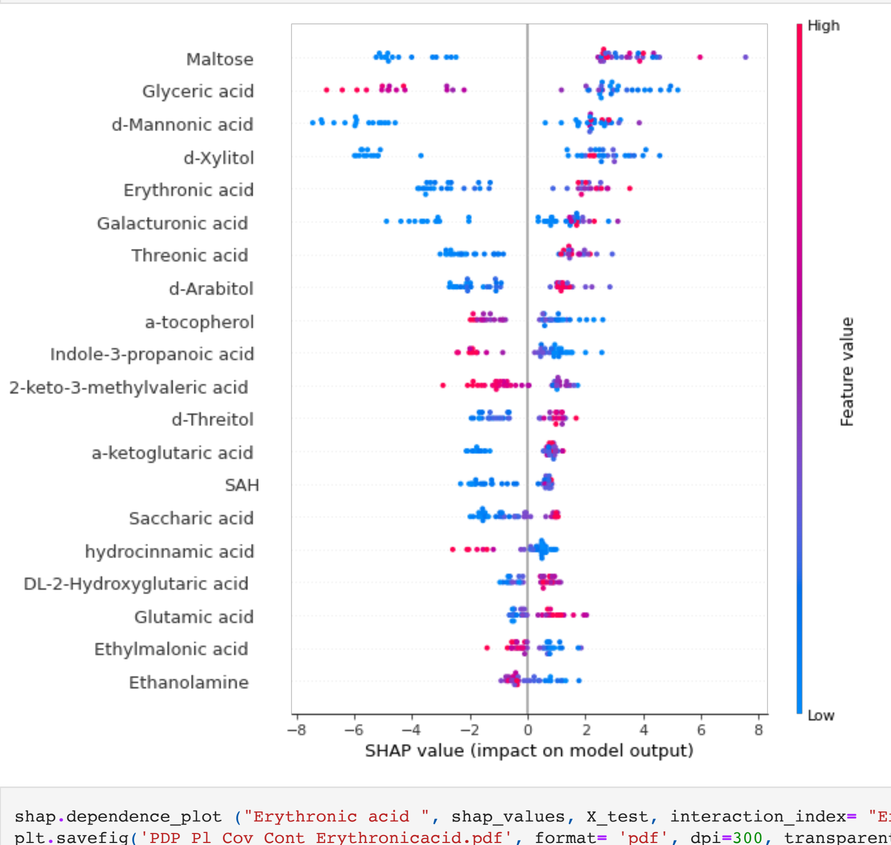

In [54]:
```python
shap.dependence_plot ("Erythronic acid ", shap_values, X_test, interaction_index= "Erythronic acid ", dot_size=20, x_jitter=3, alpha = 0.8, cmap=plt.get_cmap("cividis")
plt.savefig('PDP_Pl_Cov_Cont_Erythronicacid.pdf', format= 'pdf', dpi=300, transparent = True, bbox_inches='tight')
```

```
`should_run_async` will not call `transform_cell` automatically in the future. Please pass the result to `transformed_cell` argument and any exception that happen during thetransform in `preprocessing_exc_tuple` in IPython 7.17 and above.
```

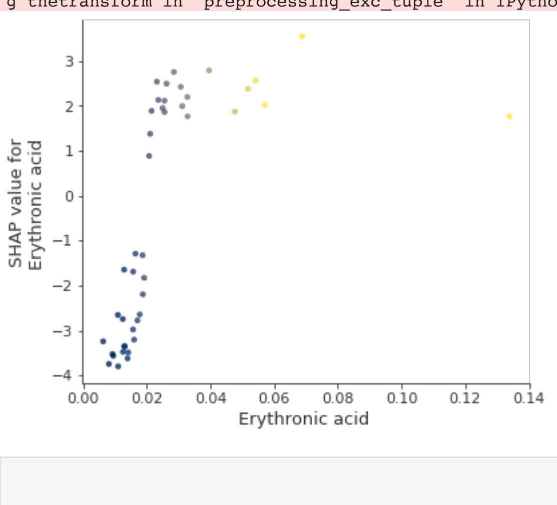

In [ ]:

In [1]:

```python
# Data treatment
# ==============================================================================
import numpy as np
import pandas as pd
import statsmodels.api as sm

# Graphs
# ==============================================================================
import matplotlib.pyplot as plt

# Processing and modeling
# ==============================================================================
from sklearn.datasets import load_boston
from sklearn.ensemble import GradientBoostingClassifier
from sklearn.metrics import accuracy_score
from sklearn.metrics import confusion_matrix
from sklearn.compose import ColumnTransformer
from sklearn.preprocessing import OneHotEncoder
from sklearn.model_selection import cross_val_score
from sklearn.model_selection import train_test_split
from sklearn.model_selection import RepeatedKFold
from sklearn.model_selection import GridSearchCV
from sklearn.model_selection import ParameterGrid
from sklearn.inspection import permutation_importance
import multiprocessing

# Warnings configuration
# ==============================================================================
import warnings
warnings.filterwarnings('once')
```

In [2]:

```python
datos = pd.read_csv("Pl_NoCov_Control.csv")
```

```
/Users/gerard/opt/anaconda3/lib/python3.8/site-packages/ipykernel/ipkernel.py:287: DeprecationWarning: `should_run_async` will not call `transform_cell` automatically i
n the future. Please pass the result to `transformed_cell` argument and any exception that happen during thetransform in `preprocessing_exc_tuple` in IPython 7.17 and a
bove.
  and should_run_async(code)
```

In [3]:

```python
# Division of data into train and test
# ==============================================================================
X_train, X_test, y_train, y_test = train_test_split(
                                        datos.drop(columns = 'Group'),
                                        datos['Group'],
                                        random_state = 123
                                    )

# One-hot-encoding of categorical variables
# ==============================================================================
# The name of the numerical and categorical columns is identified
cat_cols = X_train.select_dtypes(include=['object', 'category']).columns.to_list()
numeric_cols = X_train.select_dtypes(include=['float64', 'int']).columns.to_list()

#  One-hot-encoding is applied only to categorical columns
preprocessor = ColumnTransformer(
                    [('onehot', OneHotEncoder(handle_unknown='ignore'), cat_cols)],
                    remainder='passthrough'
               )

# Once the ColumnTransformer object has been defined, with the fit() method
# method, the transformations are learned with the training data and are applied to
# the two sets with transform(). Both operations at the same time with fit_transform().
X_train_prep = preprocessor.fit_transform(X_train)
X_test_prep  = preprocessor.transform(X_test)
```

In [4]:

```python
# Grid of evaluated hyperparameters
# ==============================================================================
param_grid = {'n_estimators'  : [50, 100, 500, 1000],
              'max_features'  : ['auto', 'sqrt', 'log2'],
              'max_depth'     : [None, 1, 3, 5, 10, 20],
              'subsample'     : [0.5, 1],
              'learning_rate' : [0.001, 0.01, 0.1]
             }

# Grid search with cross validation
# ==============================================================================
grid = GridSearchCV(
        estimator  = GradientBoostingClassifier(random_state=123),
        param_grid = param_grid,
        scoring    = 'accuracy',
        n_jobs     = multiprocessing.cpu_count() - 1,
        cv         = RepeatedKFold(n_splits=3, n_repeats=1, random_state=123),
        refit      = True,
        verbose    = 0,
        return_train_score = True
       )

grid.fit(X = X_train_prep, y = y_train)

# Results
# ==============================================================================
resultados = pd.DataFrame(grid.cv_results_)
resultados.filter(regex = '(param*|mean_t|std_t)') \
    .drop(columns = 'params') \
    .sort_values('mean_test_score', ascending = False) \
    .head(4)
```

Out[4]:

	param_learning_rate	param_max_depth	param_max_features	param_n_estimators	param_subsample	mean_test_score	std_test_score	mean_train_score	std_train_score
**224**	0.01	5	sqrt	50	0.5	0.985507	0.020496	1.0	0.0
**227**	0.01	5	sqrt	100	1	0.971014	0.020496	1.0	0.0
**249**	0.01	10	sqrt	50	1	0.971014	0.020496	1.0	0.0
**369**	0.1	5	sqrt	50	1	0.971014	0.020496	1.0	0.0

In [5]:

```python
# Better hyperparameters by cross-validation
# ==============================================================================
print("----------------------------------------")
print("Mejores hiperparámetros encontrados (cv)")
print("----------------------------------------")
print(grid.best_params_, ":", grid.best_score_, grid.scoring)
```

```
----------------------------------------
Mejores hiperparámetros encontrados (cv)
----------------------------------------
{'learning_rate': 0.01, 'max_depth': 5, 'max_features': 'sqrt', 'n_estimators': 50, 'subsample': 0.5} : 0.9855072463768115 accuracy
/Users/gerard/opt/anaconda3/lib/python3.8/site-packages/ipykernel/ipkernel.py:287: DeprecationWarning: `should_run_async` will not call `transform_cell` automatically i
n the future. Please pass the result to `transformed_cell` argument and any exception that happen during thetransform in `preprocessing_exc_tuple` in IPython 7.17 and a
bove.
  and should_run_async(code)
```

In [6]:

```python
modelo_final = grid.best_estimator_
```

In [7]:

```python
# Final model test error
# ==============================================================================
predicciones = modelo_final.predict(X = X_test_prep)
predicciones[:10]
```

Out[7]:

```
array([0, 0, 1, 0, 0, 1, 1, 0, 0, 1])
```

In [8]:

```python
mat_confusion = confusion_matrix(
                    y_true    = y_test,
                    y_pred    = predicciones
                )

accuracy = accuracy_score(
            y_true    = y_test,
            y_pred    = predicciones,
            normalize = True
           )

print("Matriz de confusión")
print("-------------------")
print(mat_confusion)
print("")
print(f"El accuracy de test es: {100 * accuracy} %")
```

```
Matriz de confusión
-------------------
[[14  1]
 [ 0  9]]

El accuracy de test es: 95.83333333333334 %
/Users/gerard/opt/anaconda3/lib/python3.8/site-packages/ipykernel/ipkernel.py:287: DeprecationWarning: `should_run_async` will not call `transform_cell` automatically i
n the future. Please pass the result to `transformed_cell` argument and any exception that happen during thetransform in `preprocessing_exc_tuple` in IPython 7.17 and a
bove.
  and should_run_async(code)
```

In [14]:

```python
import seaborn as sns
import matplotlib.pyplot as plt

ax= plt.subplot()
sns.heatmap(mat_confusion, annot=True, ax = ax, cmap=plt.cm.Reds); #annot=True to annotate cells

# labels, title and ticks
ax.set_xlabel('Predicted labels');ax.set_ylabel('True labels');
ax.set_title('Confusion Matrix');
ax.xaxis.set_ticklabels(['Control', 'NoCov']); ax.yaxis.set_ticklabels(['Control', 'NoCov']);
plt.savefig('Matrix_NoC_Cont.pdf', format= 'pdf', dpi=300, transparent = True, bbox_inches='tight')
```

```
/Users/gerard/opt/anaconda3/lib/python3.8/site-packages/ipykernel/ipkernel.py:287: DeprecationWarning: `should_run_async` will not call `transform_cell` automatically i
n the future. Please pass the result to `transformed_cell` argument and any exception that happen during thetransform in `preprocessing_exc_tuple` in IPython 7.17 and a
bove.
  and should_run_async(code)
```

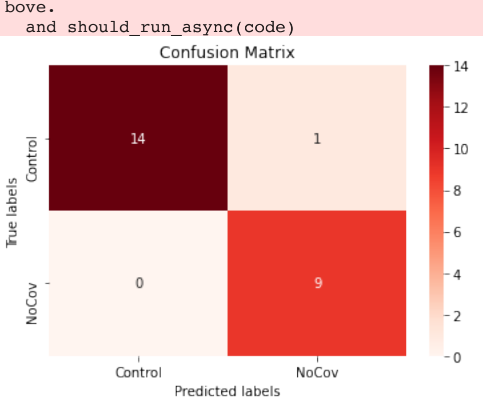

In [15]:

```python
# roc curve and auc score
from sklearn.datasets import make_classification
from sklearn.neighbors import KNeighborsClassifier
from sklearn.ensemble import RandomForestClassifier
from sklearn.model_selection import train_test_split
from sklearn.metrics import roc_curve
from sklearn.metrics import roc_auc_score

def plot_roc_curve(fpr, tpr):
    plt.plot(fpr, tpr, color='gold', label='ROC')
    plt.plot([0, 1], [0, 1], color='darkblue', linestyle='--')
    plt.xlabel('False Positive Rate')
    plt.ylabel('True Positive Rate')
    plt.title('Receiver Operating Characteristic (ROC) Curve')
    plt.legend()
    plt.savefig('AUC_Pl_NoC_Cont.pdf', format= 'pdf', dpi=300, transparent = True, bbox_inches='tight')
```

```
/Users/gerard/opt/anaconda3/lib/python3.8/site-packages/ipykernel/ipkernel.py:287: DeprecationWarning: `should_run_async` will not call `transform_cell` automatically i
n the future. Please pass the result to `transformed_cell` argument and any exception that happen during thetransform in `preprocessing_exc_tuple` in IPython 7.17 and a
bove.
  and should_run_async(code)
```

In [16]:

```python
probs = modelo_final.predict(X_test_prep)
```

In [17]:

```python
auc = roc_auc_score(y_test, probs)
print('AUC: %.2f' % auc)
```

```
AUC: 0.97
/Users/gerard/opt/anaconda3/lib/python3.8/site-packages/ipykernel/ipkernel.py:287: DeprecationWarning: `should_run_async` will not call `transform_cell` automatically i
n the future. Please pass the result to `transformed_cell` argument and any exception that happen during thetransform in `preprocessing_exc_tuple` in IPython 7.17 and a
bove.
  and should_run_async(code)
```

In [18]:

```python
fpr, tpr, thresholds = roc_curve(y_test, probs)

plot_roc_curve(fpr,tpr)
```

```
/Users/gerard/opt/anaconda3/lib/python3.8/site-packages/ipykernel/ipkernel.py:287: DeprecationWarning: `should_run_async` will not call `transform_cell` automatically i
n the future. Please pass the result to `transformed_cell` argument and any exception that happen during thetransform in `preprocessing_exc_tuple` in IPython 7.17 and a
bove.
  and should_run_async(code)
```

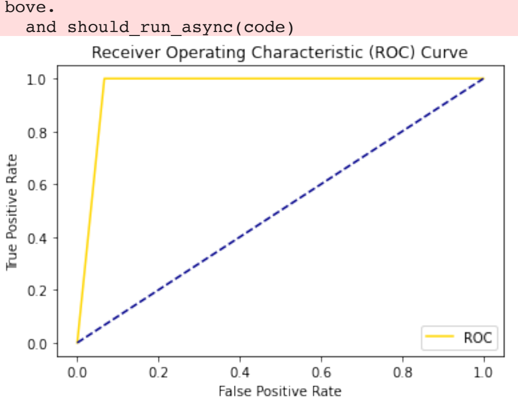

In [19]:

```python
# Probability prediction
# ==============================================================================
predicciones = modelo_final.predict_proba(X = X_test_prep)
predicciones[:5, :]
```

```
/Users/gerard/opt/anaconda3/lib/python3.8/site-packages/ipykernel/ipkernel.py:287: DeprecationWarning: `should_run_async` will not call `transform_cell` automatically i
n the future. Please pass the result to `transformed_cell` argument and any exception that happen during thetransform in `preprocessing_exc_tuple` in IPython 7.17 and a
bove.
  and should_run_async(code)
```

Out[19]:

```
array([[0.5904542 , 0.4095458 ],
       [0.69212443, 0.30787557],
       [0.40948447, 0.59051553],
       [0.69191476, 0.30808524],
       [0.55836536, 0.44163464]])
```

In [20]:

```python
# Classification using the highest probability class
# ==============================================================================
df_predicciones = pd.DataFrame(data=predicciones, columns=['0', '1'])
df_predicciones['clasificacion_default_0.5'] = np.where(df_predicciones['0'] > df_predicciones['1'], 0, 1)
df_predicciones.head(3)
```

```
/Users/gerard/opt/anaconda3/lib/python3.8/site-packages/ipykernel/ipkernel.py:287: DeprecationWarning: `should_run_async` will not call `transform_cell` automatically i
n the future. Please pass the result to `transformed_cell` argument and any exception that happen during thetransform in `preprocessing_exc_tuple` in IPython 7.17 and a
bove.
  and should_run_async(code)
```

Out[20]:

	0	1	clasificacion_default_0.5
**0**	0.590454	0.409546	0
**1**	0.692124	0.307876	0
**2**	0.409484	0.590516	1

In [21]:

```python
# Final classification using a threshold of 0.8 for class 1.
# ==============================================================================
df_predicciones['clasificacion_custom_0.8'] = np.where(df_predicciones['1'] > 0.9, 1, 0)
df_predicciones[df_predicciones['clasificacion_custom_0.8']!=df_predicciones['clasificacion_default_0.5']] \
    .head(3)
```

```
/Users/gerard/opt/anaconda3/lib/python3.8/site-packages/ipykernel/ipkernel.py:287: DeprecationWarning: `should_run_async` will not call `transform_cell` automatically i
n the future. Please pass the result to `transformed_cell` argument and any exception that happen during thetransform in `preprocessing_exc_tuple` in IPython 7.17 and a
bove.
  and should_run_async(code)
```

Out[21]:

	0	1	clasificacion_default_0.5	clasificacion_custom_0.8
**2**	0.409484	0.590516	1	0
**5**	0.377248	0.622752	1	0
**6**	0.303227	0.696773	1	0

In [22]:

```python
importancia_predictores = pd.DataFrame(
                            {'predictor': datos.drop(columns = "Group").columns,
                             'importancia': modelo_final.feature_importances_}
                            )
print("Importancia de los predictores en el modelo")
print("-------------------------------------------")
importancia_predictores.sort_values('importancia', ascending=False)
```

```
Importancia de los predictores en el modelo
-------------------------------------------
/Users/gerard/opt/anaconda3/lib/python3.8/site-packages/ipykernel/ipkernel.py:287: DeprecationWarning: `should_run_async` will not call `transform_cell` automatically i
n the future. Please pass the result to `transformed_cell` argument and any exception that happen during thetransform in `preprocessing_exc_tuple` in IPython 7.17 and a
bove.
  and should_run_async(code)
```

Out[22]:

	predictor	importancia
**17**	Phosphoric acid	1.011511e-01
**60**	d-Mannonic acid	7.771688e-02
**30**	d-Threitol	6.685318e-02
**34**	Erythronic acid	6.455771e-02
**28**	Malic acid	5.631746e-02
**...**	...	...
**53**	Citric acid	3.952965e-06
**79**	Choline	2.674726e-06
**55**	Tetradecanoic acid	1.405692e-06
**65**	Indole-3-propanoic acid	1.288848e-06
**57**	Vanillylmandelic acid	5.870208e-07

85 rows × 2 columns

In [23]:

```python
import shap
shap.initjs()
explainer = shap.TreeExplainer(modelo_final)
```

```
/Users/gerard/opt/anaconda3/lib/python3.8/site-packages/ipykernel/ipkernel.py:287: DeprecationWarning: `should_run_async` will not call `transform_cell` automatically i
n the future. Please pass the result to `transformed_cell` argument and any exception that happen during thetransform in `preprocessing_exc_tuple` in IPython 7.17 and a
bove.
  and should_run_async(code)
<frozen importlib._bootstrap>:219: RuntimeWarning: numpy.ufunc size changed, may indicate binary incompatibility. Expected 192 from C header, got 216 from PyObject
Converting `np.inexact` or `np.floating` to a dtype is deprecated. The current result is `float64` which is not strictly correct.
```

```
Setting feature_perturbation = "tree_path_dependent" because no background data was given.
The sklearn.ensemble.gradient_boosting module is  deprecated in version 0.22 and will be removed in version 0.24. The corresponding classes / functions should instead b
e imported from sklearn.ensemble. Anything that cannot be imported from sklearn.ensemble is now part of the private API.
```

In [26]:

```python
import numpy as np
import matplotlib as mpl
import matplotlib.pyplot as plt
from matplotlib import cm

from collections import OrderedDict

shap.summary_plot(shap_values, X_test,plot_type="bar", max_display= 5, show= False)
plt.savefig('SHAPC_NoC_Cont.pdf', format= 'pdf', dpi=300, transparent = True, bbox_inches='tight')
```

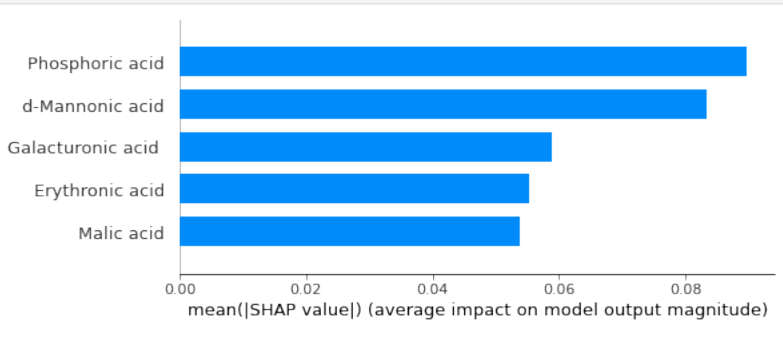

In [25]:

```python
shap_values = explainer.shap_values(X_test, approximate=False, check_additivity=False)

shap.summary_plot(shap_values, X_test)
```

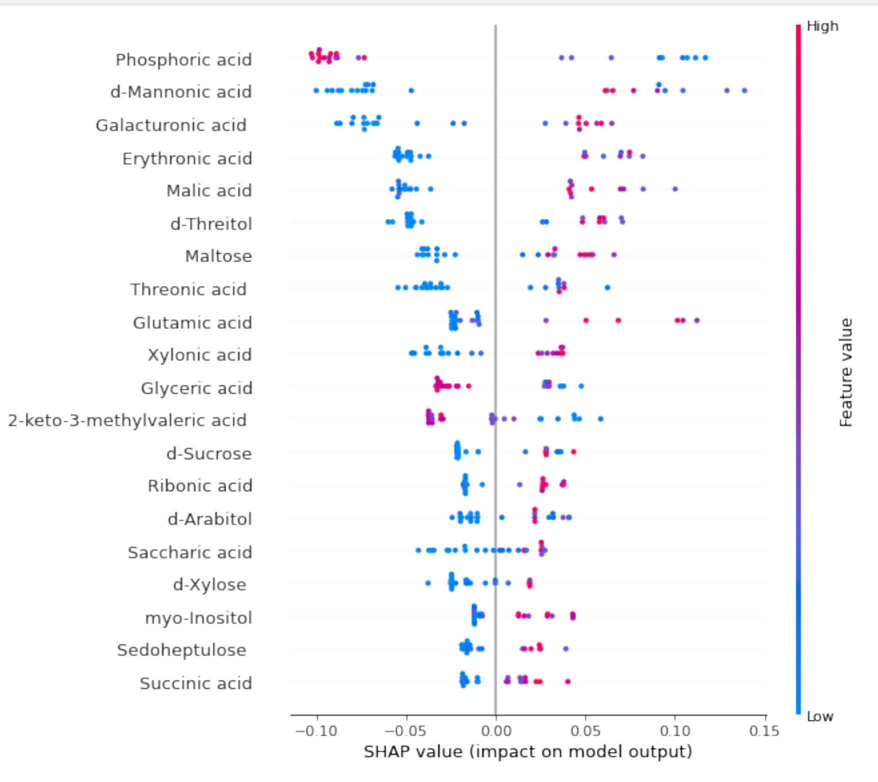

In [36]:

```python
shap.dependence_plot ('Malic acid ', shap_values, X_test, interaction_index= "Malic acid ", dot_size=20, x_jitter=3, alpha = 0.8, cmap=plt.get_cmap("cividis"), show=Fa
plt.savefig('PDP_Pl_Malic acid.pdf', format= 'pdf', dpi=300, transparent = True, bbox_inches='tight')
```

```
`should_run_async` will not call `transform_cell` automatically in the future. Please pass the result to `transformed_cell` argument and any exception that happen durin
g thetransform in `preprocessing_exc_tuple` in IPython 7.17 and above.
```

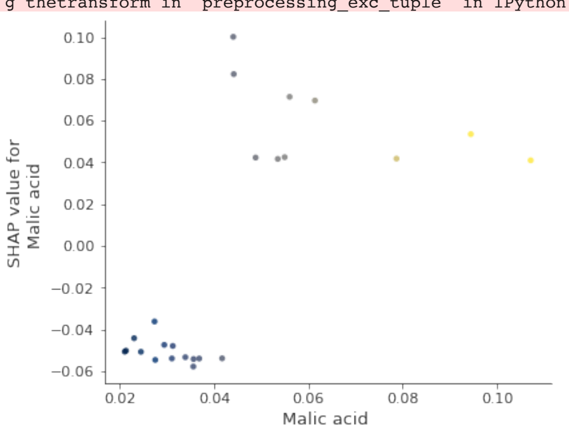

In [ ]:

In [1]:

```
# Data treatment
# ==============================================================================
import numpy as np
import pandas as pd
import statsmodels.api as sm

# Graphs
# ==============================================================================
import matplotlib.pyplot as plt

# Processing and modeling
# ==============================================================================
from sklearn.datasets import load_boston
from sklearn.ensemble import GradientBoostingClassifier
from sklearn.metrics import accuracy_score
from sklearn.metrics import confusion_matrix
from sklearn.compose import ColumnTransformer
from sklearn.preprocessing import OneHotEncoder
from sklearn.model_selection import cross_val_score
from sklearn.model_selection import train_test_split
from sklearn.model_selection import RepeatedKFold
from sklearn.model_selection import GridSearchCV
from sklearn.model_selection import ParameterGrid
from sklearn.inspection import permutation_importance
import multiprocessing

# Warnings configuration
# ==============================================================================
import warnings
warnings.filterwarnings('once')
```

In [2]:

```
datos = pd.read_csv("Covid_Nocovid_Pl.csv")
```

```
/Users/gerard/opt/anaconda3/lib/python3.8/site-packages/ipykernel/ipkernel.py:287: DeprecationWarning: `should_run_async` will not call `transform_cell` automatically i
n the future. Please pass the result to `transformed_cell` argument and any exception that happen during thetransform in `preprocessing_exc_tuple` in IPython 7.17 and a
bove.
  and should_run_async(code)
```

In [3]:

```
# Division of data into train and test
# ==============================================================================
X_train, X_test, y_train, y_test = train_test_split(
                                        datos.drop(columns = 'Group'),
                                        datos['Group'],
                                        random_state = 123
                                    )

# One-hot-encoding of categorical variables
# ==============================================================================
# The name of the numerical and categorical columns is identified
cat_cols = X_train.select_dtypes(include=['object', 'category']).columns.to_list()
numeric_cols = X_train.select_dtypes(include=['float64', 'int']).columns.to_list()

# One-hot-encoding is applied only to categorical columns
preprocessor = ColumnTransformer(
                    [('onehot', OneHotEncoder(handle_unknown='ignore'), cat_cols)],
                    remainder='passthrough'
                )

# Once the ColumnTransformer object has been defined, with the fit() method
# method, the transformations are learned with the training data and are applied to
# the two sets with transform(). Both operations at the same time with fit_transform().
X_train_prep = preprocessor.fit_transform(X_train)
X_test_prep  = preprocessor.transform(X_test)
```

In [4]:

```
# Grid of evaluated hyperparameters
# ==============================================================================
param_grid = {'n_estimators'  : [50, 100, 500, 1000],
              'max_features'  : ['auto', 'sqrt', 'log2'],
              'max_depth'     : [None, 1, 3, 5, 10, 20],
              'subsample'     : [0.5, 1],
              'learning_rate' : [0.001, 0.01, 0.1]
             }

# Grid search with cross validation
# ==============================================================================
grid = GridSearchCV(
        estimator  = GradientBoostingClassifier(random_state=123),
        param_grid = param_grid,
        scoring    = 'accuracy',
        n_jobs     = multiprocessing.cpu_count() - 1,
        cv         = RepeatedKFold(n_splits=3, n_repeats=1, random_state=123),
        refit      = True,
        verbose    = 0,
        return_train_score = True
       )

grid.fit(X = X_train_prep, y = y_train)

# Results
# ==============================================================================
resultados = pd.DataFrame(grid.cv_results_)
resultados.filter(regex = '(param*|mean_t|std_t)') \
    .drop(columns = 'params') \
    .sort_values('mean_test_score', ascending = False) \
    .head(4)
```

Out[4]:

	param_learning_rate	param_max_depth	param_max_features	param_n_estimators	param_subsample	mean_test_score	std_test_score	mean_train_score	std_train_score
**351**	0.1	3	sqrt	1000	1	0.909350	0.043132	1.0	0.0
**359**	0.1	3	log2	1000	1	0.909350	0.043132	1.0	0.0
**375**	0.1	5	sqrt	1000	1	0.909146	0.062745	1.0	0.0
**429**	0.1	20	log2	500	1	0.901423	0.021171	1.0	0.0

In [5]:

```
# Better hyperparameters by cross-validation
# ==============================================================================
print("----------------------------------------")
print("Mejores hiperparámetros encontrados (cv)")
print("----------------------------------------")
print(grid.best_params_, ":", grid.best_score_, grid.scoring)
```

```
----------------------------------------
Mejores hiperparámetros encontrados (cv)
----------------------------------------
{'learning_rate': 0.1, 'max_depth': 3, 'max_features': 'sqrt', 'n_estimators': 1000, 'subsample': 1} : 0.9093495934959349 accuracy
/Users/gerard/opt/anaconda3/lib/python3.8/site-packages/ipykernel/ipkernel.py:287: DeprecationWarning: `should_run_async` will not call `transform_cell` automatically i
n the future. Please pass the result to `transformed_cell` argument and any exception that happen during thetransform in `preprocessing_exc_tuple` in IPython 7.17 and a
bove.
  and should_run_async(code)
```

In [6]:

```
modelo_final = grid.best_estimator_
```

In [7]:

```
# Final model test error
# ==============================================================================
predicciones = modelo_final.predict(X = X_test_prep)
predicciones[:10]
```

Out[7]:

```
array([0, 1, 1, 0, 1, 1, 1, 0, 1, 0])
```

In [8]:

```
mat_confusion = confusion_matrix(
                    y_true    = y_test,
                    y_pred    = predicciones
                )

accuracy = accuracy_score(
            y_true    = y_test,
            y_pred    = predicciones,
            normalize = True
           )

print("Matriz de confusión")
print("-------------------")
print(mat_confusion)
print("")
print(f"El accuracy de test es: {100 * accuracy} %")
```

```
Matriz de confusión
-------------------
[[ 9  2]
 [ 1 29]]

El accuracy de test es: 92.6829268292683 %
/Users/gerard/opt/anaconda3/lib/python3.8/site-packages/ipykernel/ipkernel.py:287: DeprecationWarning: `should_run_async` will not call `transform_cell` automatically i
n the future. Please pass the result to `transformed_cell` argument and any exception that happen during thetransform in `preprocessing_exc_tuple` in IPython 7.17 and a
bove.
  and should_run_async(code)
```

In [9]:

```
import seaborn as sns
import matplotlib.pyplot as plt

ax= plt.subplot()
sns.heatmap(mat_confusion, annot=True, ax = ax, cmap=plt.cm.Reds); #annot=True to annotate cells

# labels, title and ticks
ax.set_xlabel('Predicted labels');ax.set_ylabel('True labels');
ax.set_title('Confusion Matrix');
ax.xaxis.set_ticklabels(['No-Covid', 'Covid']); ax.yaxis.set_ticklabels(['No-Covid', 'Covid']);
plt.savefig('Matrix_Pl_Cov_NoCov.pdf', format= 'pdf', dpi=300, transparent = True, bbox_inches='tight')
```

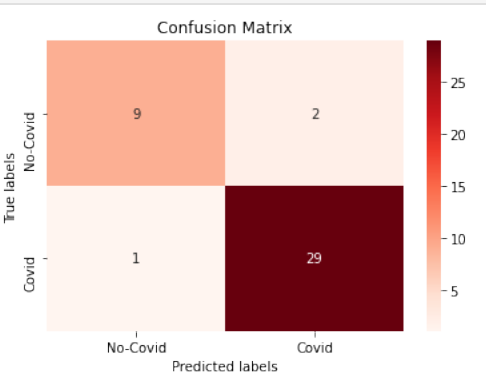

In [14]:

```
# roc curve and auc score
from sklearn.datasets import make_classification
from sklearn.neighbors import KNeighborsClassifier
from sklearn.ensemble import RandomForestClassifier
from sklearn.model_selection import train_test_split
from sklearn.metrics import roc_curve
from sklearn.metrics import roc_auc_score

def plot_roc_curve(fpr, tpr):
    plt.plot(fpr, tpr, color='gold', label='ROC')
    plt.plot([0, 1], [0, 1], color='darkblue', linestyle='--')
    plt.xlabel('False Positive Rate')
    plt.ylabel('True Positive Rate')
    plt.title('Receiver Operating Characteristic (ROC) Curve')
    plt.legend()
    plt.savefig('AUC_Pl_Cov_NoCov.pdf', format= 'pdf', dpi=300, transparent = True, bbox_inches='tight')
```

```
/Users/gerard/opt/anaconda3/lib/python3.8/site-packages/ipykernel/ipkernel.py:287: DeprecationWarning: `should_run_async` will not call `transform_cell` automatically i
n the future. Please pass the result to `transformed_cell` argument and any exception that happen during thetransform in `preprocessing_exc_tuple` in IPython 7.17 and a
bove.
  and should_run_async(code)
```

In [15]:

```
probs = modelo_final.predict(X_test_prep)
```

In [16]:

```
auc = roc_auc_score(y_test, probs)
print('AUC: %.2f' % auc)
```

```
AUC: 0.89
/Users/gerard/opt/anaconda3/lib/python3.8/site-packages/ipykernel/ipkernel.py:287: DeprecationWarning: `should_run_async` will not call `transform_cell` automatically i
n the future. Please pass the result to `transformed_cell` argument and any exception that happen during thetransform in `preprocessing_exc_tuple` in IPython 7.17 and a
bove.
  and should_run_async(code)
```

In [17]:

```
fpr, tpr, thresholds = roc_curve(y_test, probs)

plot_roc_curve(fpr,tpr)
```

```
/Users/gerard/opt/anaconda3/lib/python3.8/site-packages/ipykernel/ipkernel.py:287: DeprecationWarning: `should_run_async` will not call `transform_cell` automatically i
n the future. Please pass the result to `transformed_cell` argument and any exception that happen during thetransform in `preprocessing_exc_tuple` in IPython 7.17 and a
bove.
  and should_run_async(code)
```

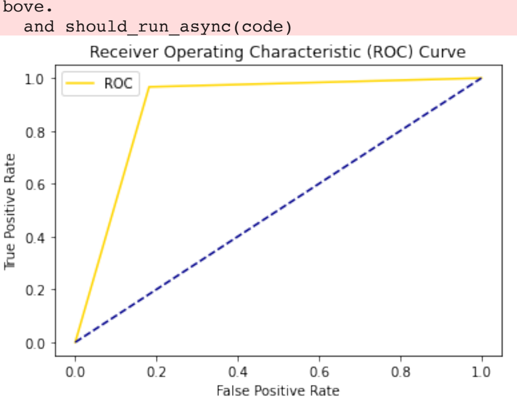

In [18]:

```
# Predicción de probabilidades
# ==============================================================================
predicciones = modelo_final.predict_proba(X = X_test_prep)
predicciones[:5, :]
```

```
/Users/gerard/opt/anaconda3/lib/python3.8/site-packages/ipykernel/ipkernel.py:287: DeprecationWarning: `should_run_async` will not call `transform_cell` automatically i
n the future. Please pass the result to `transformed_cell` argument and any exception that happen during thetransform in `preprocessing_exc_tuple` in IPython 7.17 and a
bove.
  and should_run_async(code)
```

Out[18]:

```
array([[1.00000000e+00, 5.24749624e-11],
       [9.61994928e-11, 1.00000000e+00],
       [5.99520433e-15, 1.00000000e+00],
       [1.00000000e+00, 4.55878498e-10],
       [5.17472509e-10, 9.99999999e-01]])
```

In [19]:

```
# Clasificación empleando la clase de mayor probabilidad
# ==============================================================================
df_predicciones = pd.DataFrame(data=predicciones, columns=['0', '1'])
df_predicciones['clasificacion_default_0.5'] = np.where(df_predicciones['0'] > df_predicciones['1'], 0, 1)
df_predicciones.head(3)
```

```
/Users/gerard/opt/anaconda3/lib/python3.8/site-packages/ipykernel/ipkernel.py:287: DeprecationWarning: `should_run_async` will not call `transform_cell` automatically i
n the future. Please pass the result to `transformed_cell` argument and any exception that happen during thetransform in `preprocessing_exc_tuple` in IPython 7.17 and a
bove.
  and should_run_async(code)
```

Out[19]:

	0	1	clasificacion_default_0.5
**0**	1.000000e+00	5.247496e-11	0
**1**	9.619949e-11	1.000000e+00	1
**2**	5.995204e-15	1.000000e+00	1

In [20]:

```
# Clasificación final empleando un threshold de 0.8 para la clase 1.
# ==============================================================================
df_predicciones['clasificacion_custom_0.8'] = np.where(df_predicciones['1'] > 0.9, 1, 0)
df_predicciones[df_predicciones['clasificacion_custom_0.8']!=df_predicciones['clasificacion_default_0.5']] \
    .head(3)
```

```
/Users/gerard/opt/anaconda3/lib/python3.8/site-packages/ipykernel/ipkernel.py:287: DeprecationWarning: `should_run_async` will not call `transform_cell` automatically i
n the future. Please pass the result to `transformed_cell` argument and any exception that happen during thetransform in `preprocessing_exc_tuple` in IPython 7.17 and a
bove.
  and should_run_async(code)
```

Out[20]:

	0	1	clasificacion_default_0.5	clasificacion_custom_0.8
**19**	0.311844	0.688156	1	0

In [21]:

```
importancia_predictores = pd.DataFrame(
                            {'predictor': datos.drop(columns = "Group").columns,
                             'importancia': modelo_final.feature_importances_}
                            )
print("Importancia de los predictores en el modelo")
print("-------------------------------------------")
importancia_predictores.sort_values('importancia', ascending=False)
```

```
Importancia de los predictores en el modelo
-------------------------------------------
/Users/gerard/opt/anaconda3/lib/python3.8/site-packages/ipykernel/ipkernel.py:287: DeprecationWarning: `should_run_async` will not call `transform_cell` automatically i
n the future. Please pass the result to `transformed_cell` argument and any exception that happen during thetransform in `preprocessing_exc_tuple` in IPython 7.17 and a
bove.
  and should_run_async(code)
```

Out[21]:

	predictor	importancia
**21**	Succinic acid	1.649431e-01
**38**	4-Hydroxybenzoic acid	7.853482e-02
**17**	Phosphoric acid	6.516502e-02
**50**	Hypoxanthine	6.444700e-02
**83**	SAH	5.808688e-02
**...**	...	...
**2**	2-Hydroxyisobutyric acid	4.881472e-06
**65**	Indole-3-propanoic acid	3.877914e-06
**80**	Dimethylglycine	3.223251e-06
**10**	3-Hydroxyisovaleric acid	7.302245e-07
**33**	Threonic acid	3.492353e-12

85 rows × 2 columns

In [22]:

```
import shap
shap.initjs()
explainer = shap.TreeExplainer(modelo_final)
```

```
/Users/gerard/opt/anaconda3/lib/python3.8/site-packages/ipykernel/ipkernel.py:287: DeprecationWarning: `should_run_async` will not call `transform_cell` automatically i
n the future. Please pass the result to `transformed_cell` argument and any exception that happen during thetransform in `preprocessing_exc_tuple` in IPython 7.17 and a
bove.
  and should_run_async(code)
<frozen importlib._bootstrap>:219: RuntimeWarning: numpy.ufunc size changed, may indicate binary incompatibility. Expected 192 from C header, got 216 from PyObject
Converting `np.inexact` or `np.floating` to a dtype is deprecated. The current result is `float64` which is not strictly correct.
```

```
Setting feature_perturbation = "tree_path_dependent" because no background data was given.
The sklearn.ensemble.gradient_boosting module is  deprecated in version 0.22 and will be removed in version 0.24. The corresponding classes / functions should instead b
e imported from sklearn.ensemble. Anything that cannot be imported from sklearn.ensemble is now part of the private API.
```

In [25]:

```
import numpy as np
import matplotlib as mpl
import matplotlib.pyplot as plt
from matplotlib import cm

from collections import OrderedDict

shap.summary_plot(shap_values, X_test,plot_type="bar", max_display= 5, show= False)
plt.savefig('SHAPC_Pl_Cov_NoCov.pdf', format= 'pdf', dpi=300, transparent = True, bbox_inches='tight')
```

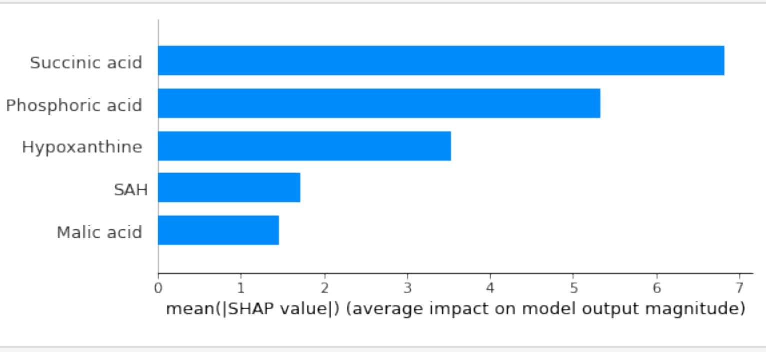

In [24]:

```
shap_values = explainer.shap_values(X_test, approximate=False, check_additivity=False)

shap.summary_plot(shap_values, X_test)
```

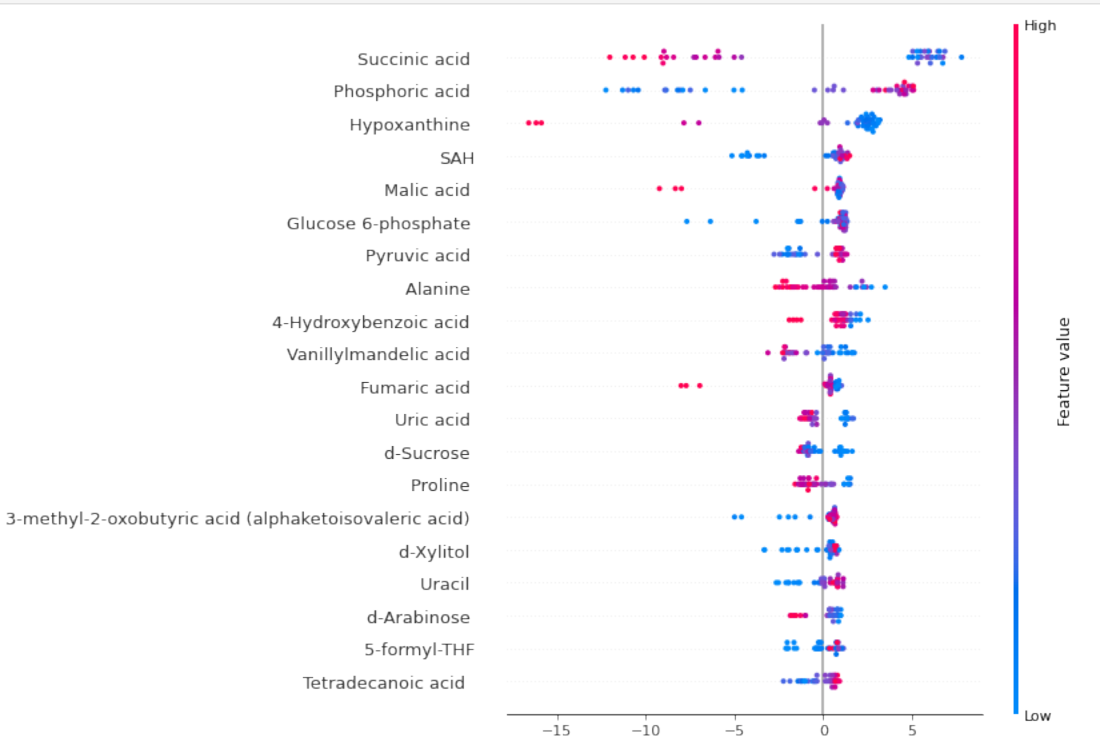

In [36]:

```
shap.dependence_plot ('Succinic acid ', shap_values, X_test, interaction_index= "Succinic acid ", dot_size=20, x_jitter=3, alpha = 0.8, cmap=plt.get_cmap("cividis"), sh
plt.savefig('PDP_Succinic acid.pdf', format= 'pdf', dpi=300, transparent = True, bbox_inches='tight')
```

```
`should_run_async` will not call `transform_cell` automatically in the future. Please pass the result to `transformed_cell` argument and any exception that happen durin
g thetransform in `preprocessing_exc_tuple` in IPython 7.17 and above.
```

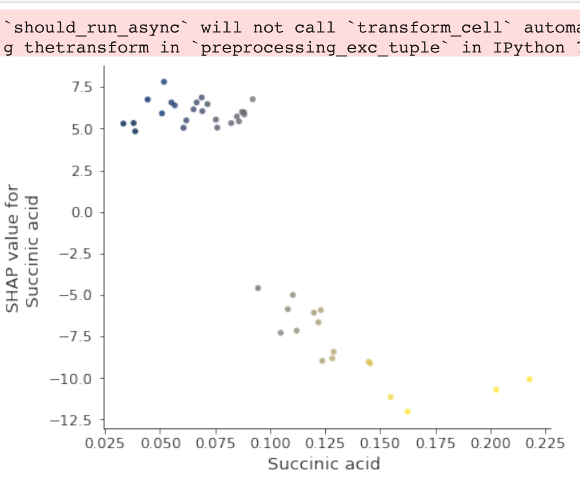

In [ ]:

In [1]:
```python
# Data treatment
# ==============================================================================
import numpy as np
import pandas as pd
import statsmodels.api as sm

# Graphs
# ==============================================================================
import matplotlib.pyplot as plt

# Processing and modeling
# ==============================================================================
from sklearn.datasets import load_boston
from sklearn.ensemble import GradientBoostingClassifier
from sklearn.metrics import accuracy_score
from sklearn.metrics import confusion_matrix
from sklearn.compose import ColumnTransformer
from sklearn.preprocessing import OneHotEncoder
from sklearn.model_selection import cross_val_score
from sklearn.model_selection import train_test_split
from sklearn.model_selection import RepeatedKFold
from sklearn.model_selection import GridSearchCV
from sklearn.model_selection import ParameterGrid
from sklearn.inspection import permutation_importance
import multiprocessing

# Warnings configuration
# ==============================================================================
import warnings
warnings.filterwarnings('once')
```

In [2]:
```python
datos = pd.read_csv("Urine_Cov_NoC.csv")
```

```
/Users/gerard/opt/anaconda3/lib/python3.8/site-packages/ipykernel/ipkernel.py:287: DeprecationWarning: `should_run_async` will not call `transform_cell` automatically i
n the future. Please pass the result to `transformed_cell` argument and any exception that happen during thetransform in `preprocessing_exc_tuple` in IPython 7.17 and a
bove.
  and should_run_async(code)
```

In [3]:
```python
# Division of data into train and test
# ==============================================================================
X_train, X_test, y_train, y_test = train_test_split(
                                        datos.drop(columns = 'Group'),
                                        datos['Group'],
                                        random_state = 123
                                    )

# One-hot-encoding of categorical variables
# ==============================================================================
# The name of the numerical and categorical columns is identified
cat_cols = X_train.select_dtypes(include=['object', 'category']).columns.to_list()
numeric_cols = X_train.select_dtypes(include=['float64', 'int']).columns.to_list()

# One-hot-encoding is applied only to categorical columns
preprocessor = ColumnTransformer(
                    [('onehot', OneHotEncoder(handle_unknown='ignore'), cat_cols)],
                    remainder='passthrough'
               )

# Once the ColumnTransformer object has been defined, with the fit() method
# method, the transformations are learned with the training data and are applied to
# the two sets with transform(). Both operations at the same time with fit_transform().
X_train_prep = preprocessor.fit_transform(X_train)
X_test_prep  = preprocessor.transform(X_test)
```

In [4]:
```python
# Grid of evaluated hyperparameters
# ==============================================================================
param_grid = {'n_estimators'  : [50, 100, 500, 1000],
              'max_features'  : ['auto', 'sqrt', 'log2'],
              'max_depth'     : [None, 1, 3, 5, 10, 20],
              'subsample'     : [0.5, 1],
              'learning_rate' : [0.001, 0.01, 0.1]
             }

# Grid search with cross validation
# ==============================================================================
grid = GridSearchCV(
        estimator  = GradientBoostingClassifier(random_state=123),
        param_grid = param_grid,
        scoring    = 'accuracy',
        n_jobs     = multiprocessing.cpu_count() - 1,
        cv         = RepeatedKFold(n_splits=3, n_repeats=1, random_state=123),
        refit      = True,
        verbose    = 0,
        return_train_score = True
       )

grid.fit(X = X_train_prep, y = y_train)

# Results
# ==============================================================================
resultados = pd.DataFrame(grid.cv_results_)
resultados.filter(regex = '(param*|mean_t|std_t)') \
    .drop(columns = 'params') \
    .sort_values('mean_test_score', ascending = False) \
    .head(4)
```

Out[4]:

	param_learning_rate	param_max_depth	param_max_features	param_n_estimators	param_subsample	mean_test_score	std_test_score	mean_train_score	std_train_score
**351**	0.1	3	sqrt	1000	1	0.796748	0.011498	1.0	0.0
**327**	0.1	1	sqrt	1000	1	0.796748	0.011498	1.0	0.0
**349**	0.1	3	sqrt	500	1	0.788618	0.022995	1.0	0.0
**301**	0.1	None	sqrt	500	1	0.788618	0.011498	1.0	0.0

In [5]:
```python
# Better hyperparameters by cross-validation
# ==============================================================================
print("----------------------------------------")
print("Mejores hiperparámetros encontrados (cv)")
print("----------------------------------------")
print(grid.best_params_, ":", grid.best_score_, grid.scoring)
```

```
----------------------------------------
Mejores hiperparámetros encontrados (cv)
----------------------------------------
{'learning_rate': 0.1, 'max_depth': 1, 'max_features': 'sqrt', 'n_estimators': 1000, 'subsample': 1} : 0.7967479674796749 accuracy
/Users/gerard/opt/anaconda3/lib/python3.8/site-packages/ipykernel/ipkernel.py:287: DeprecationWarning: `should_run_async` will not call `transform_cell` automatically i
n the future. Please pass the result to `transformed_cell` argument and any exception that happen during thetransform in `preprocessing_exc_tuple` in IPython 7.17 and a
bove.
  and should_run_async(code)
```

In [6]:
```python
modelo_final = grid.best_estimator_
```

In [7]:
```python
# Final model test error
# ==============================================================================
predicciones = modelo_final.predict(X = X_test_prep)
predicciones[:10]
```

Out[7]:
```
array([1, 1, 1, 1, 1, 1, 1, 1, 1, 1])
```

In [8]:
```python
mat_confusion = confusion_matrix(
                    y_true    = y_test,
                    y_pred    = predicciones
                )

accuracy = accuracy_score(
            y_true    = y_test,
            y_pred    = predicciones,
            normalize = True
           )

print("Matriz de confusión")
print("-------------------")
print(mat_confusion)
print("")
print(f"El accuracy de test es: {100 * accuracy} %")
```

```
Matriz de confusión
-------------------
[[ 4 11]
 [ 4 23]]

El accuracy de test es: 64.28571428571429 %
/Users/gerard/opt/anaconda3/lib/python3.8/site-packages/ipykernel/ipkernel.py:287: DeprecationWarning: `should_run_async` will not call `transform_cell` automatically i
n the future. Please pass the result to `transformed_cell` argument and any exception that happen during thetransform in `preprocessing_exc_tuple` in IPython 7.17 and a
bove.
  and should_run_async(code)
```

In [9]:
```python
import seaborn as sns
import matplotlib.pyplot as plt

ax= plt.subplot()
sns.heatmap(mat_confusion, annot=True, ax = ax, cmap=plt.cm.Reds); #annot=True to annotate cells

# labels, title and ticks
ax.set_xlabel('Predicted labels');ax.set_ylabel('True labels');
ax.set_title('Confusion Matrix');
ax.xaxis.set_ticklabels(['NoCov', 'Covid']); ax.yaxis.set_ticklabels(['NoCov', 'Covid']);
plt.savefig('Matrix_Urine_Cov_NoCov.pdf', format= 'pdf', dpi=300, transparent = True, bbox_inches='tight')
```

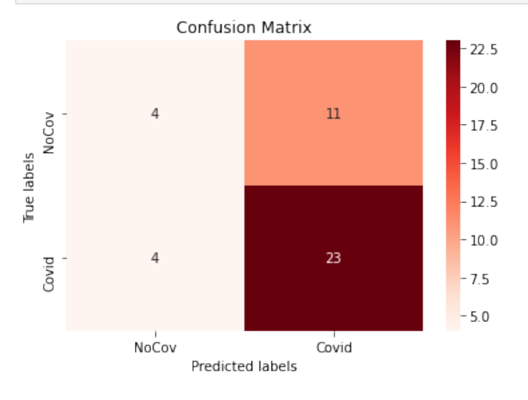

In [10]:
```python
# roc curve and auc score
from sklearn.datasets import make_classification
from sklearn.neighbors import KNeighborsClassifier
from sklearn.ensemble import RandomForestClassifier
from sklearn.model_selection import train_test_split
from sklearn.metrics import roc_curve
from sklearn.metrics import roc_auc_score

def plot_roc_curve(fpr, tpr):
    plt.plot(fpr, tpr, color='gold', label='ROC')
    plt.plot([0, 1], [0, 1], color='darkblue', linestyle='--')
    plt.xlabel('False Positive Rate')
    plt.ylabel('True Positive Rate')
    plt.title('Receiver Operating Characteristic (ROC) Curve')
    plt.legend()
    plt.savefig('AUC_Urine_Cov_NoCov.pdf', format= 'pdf', dpi=300, transparent = True, bbox_inches='tight')
```

```
/Users/gerard/opt/anaconda3/lib/python3.8/site-packages/ipykernel/ipkernel.py:287: DeprecationWarning: `should_run_async` will not call `transform_cell` automatically i
n the future. Please pass the result to `transformed_cell` argument and any exception that happen during thetransform in `preprocessing_exc_tuple` in IPython 7.17 and a
bove.
  and should_run_async(code)
```

In [11]:
```python
probs = modelo_final.predict(X_test_prep)
```

In [12]:
```python
auc = roc_auc_score(y_test, probs)
print('AUC: %.2f' % auc)
```

```
AUC: 0.56
/Users/gerard/opt/anaconda3/lib/python3.8/site-packages/ipykernel/ipkernel.py:287: DeprecationWarning: `should_run_async` will not call `transform_cell` automatically i
n the future. Please pass the result to `transformed_cell` argument and any exception that happen during thetransform in `preprocessing_exc_tuple` in IPython 7.17 and a
bove.
  and should_run_async(code)
```

In [13]:
```python
fpr, tpr, thresholds = roc_curve(y_test, probs)

plot_roc_curve(fpr,tpr)
```

```
/Users/gerard/opt/anaconda3/lib/python3.8/site-packages/ipykernel/ipkernel.py:287: DeprecationWarning: `should_run_async` will not call `transform_cell` automatically i
n the future. Please pass the result to `transformed_cell` argument and any exception that happen during thetransform in `preprocessing_exc_tuple` in IPython 7.17 and a
bove.
  and should_run_async(code)
```

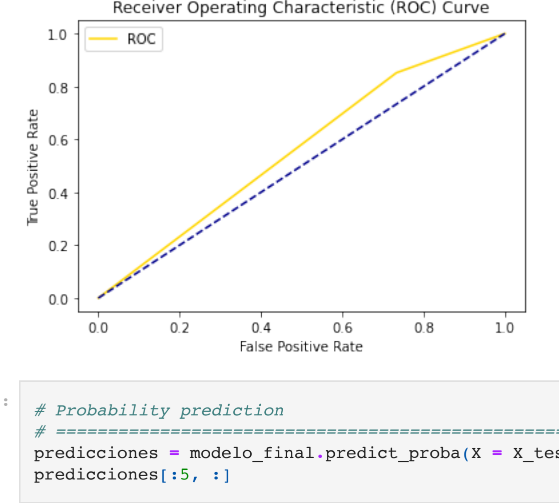

In [14]:
```python
# Probability prediction
# ==============================================================================
predicciones = modelo_final.predict_proba(X = X_test_prep)
predicciones[:5, :]
```

```
/Users/gerard/opt/anaconda3/lib/python3.8/site-packages/ipykernel/ipkernel.py:287: DeprecationWarning: `should_run_async` will not call `transform_cell` automatically i
n the future. Please pass the result to `transformed_cell` argument and any exception that happen during thetransform in `preprocessing_exc_tuple` in IPython 7.17 and a
bove.
  and should_run_async(code)
```

Out[14]:
```
array([[2.77570622e-03, 9.97224294e-01],
       [1.11222600e-04, 9.99888777e-01],
       [6.20338300e-03, 9.93796617e-01],
       [2.30550815e-03, 9.97694492e-01],
       [2.41998045e-02, 9.75800196e-01]])
```

In [15]:
```python
# Classification using the highest probability class
# ==============================================================================
df_predicciones = pd.DataFrame(data=predicciones, columns=['0', '1'])
df_predicciones['clasificacion_default_0.5'] = np.where(df_predicciones['0'] > df_predicciones['1'], 0, 1)
df_predicciones.head(3)
```

```
/Users/gerard/opt/anaconda3/lib/python3.8/site-packages/ipykernel/ipkernel.py:287: DeprecationWarning: `should_run_async` will not call `transform_cell` automatically i
n the future. Please pass the result to `transformed_cell` argument and any exception that happen during thetransform in `preprocessing_exc_tuple` in IPython 7.17 and a
bove.
  and should_run_async(code)
```

Out[15]:

	0	1	clasificacion_default_0.5
**0**	0.002776	0.997224	1
**1**	0.000111	0.999889	1
**2**	0.006203	0.993797	1

In [16]:
```python
# Final classification using a threshold of 0.8 for class 1.
# ==============================================================================
df_predicciones['clasificacion_custom_0.8'] = np.where(df_predicciones['1'] > 0.9, 1, 0)
df_predicciones[df_predicciones['clasificacion_custom_0.8']!=df_predicciones['clasificacion_default_0.5']] \
    .head(3)
```

```
/Users/gerard/opt/anaconda3/lib/python3.8/site-packages/ipykernel/ipkernel.py:287: DeprecationWarning: `should_run_async` will not call `transform_cell` automatically i
n the future. Please pass the result to `transformed_cell` argument and any exception that happen during thetransform in `preprocessing_exc_tuple` in IPython 7.17 and a
bove.
  and should_run_async(code)
```

Out[16]:

	0	1	clasificacion_default_0.5	clasificacion_custom_0.8
**11**	0.497337	0.502663	1	0
**17**	0.300714	0.699286	1	0
**18**	0.303079	0.696921	1	0

In [17]:
```python
importancia_predictores = pd.DataFrame(
                            {'predictor': datos.drop(columns = "Group").columns,
                             'importancia': modelo_final.feature_importances_}
                            )
print("Importancia de los predictores en el modelo")
print("-------------------------------------------")
importancia_predictores.sort_values('importancia', ascending=False)
```

```
Importancia de los predictores en el modelo
-------------------------------------------
/Users/gerard/opt/anaconda3/lib/python3.8/site-packages/ipykernel/ipkernel.py:287: DeprecationWarning: `should_run_async` will not call `transform_cell` automatically i
n the future. Please pass the result to `transformed_cell` argument and any exception that happen during thetransform in `preprocessing_exc_tuple` in IPython 7.17 and a
bove.
  and should_run_async(code)
```

Out[17]:

	predictor	importancia
**82**	Quinic acid	0.097172
**1**	Lactic acid	0.066604
**21**	Glycerol	0.065930
**96**	Galacturonic acid	0.065842
**72**	Ribonic acid	0.064456
**...**	...	...
**58**	d-Ribose	0.000000
**60**	d-Xylitol	0.000000
**62**	d-Arabitol	0.000000
**69**	Glutamine	0.000000
**54**	d-Xylose	0.000000

109 rows × 2 columns

In [18]:
```python
import shap
shap.initjs()
explainer = shap.TreeExplainer(modelo_final)
```

```
/Users/gerard/opt/anaconda3/lib/python3.8/site-packages/ipykernel/ipkernel.py:287: DeprecationWarning: `should_run_async` will not call `transform_cell` automatically i
n the future. Please pass the result to `transformed_cell` argument and any exception that happen during thetransform in `preprocessing_exc_tuple` in IPython 7.17 and a
bove.
  and should_run_async(code)
<frozen importlib._bootstrap>:219: RuntimeWarning: numpy.ufunc size changed, may indicate binary incompatibility. Expected 192 from C header, got 216 from PyObject
Converting `np.inexact` or `np.floating` to a dtype is deprecated. The current result is `float64` which is not strictly correct.
```

```
Setting feature_perturbation = "tree_path_dependent" because no background data was given.
The sklearn.ensemble.gradient_boosting module is  deprecated in version 0.22 and will be removed in version 0.24. The corresponding classes / functions should instead b
e imported from sklearn.ensemble. Anything that cannot be imported from sklearn.ensemble is now part of the private API.
```

In [23]:
```python
import numpy as np
import matplotlib as mpl
import matplotlib.pyplot as plt
from matplotlib import cm

from collections import OrderedDict

shap.summary_plot(shap_values, X_test,plot_type="bar", max_display= 5, show= False)
plt.savefig('SHAP_Urine_Cov_NoCov.pdf', format= 'pdf', dpi=300, transparent = True, bbox_inches='tight')
```

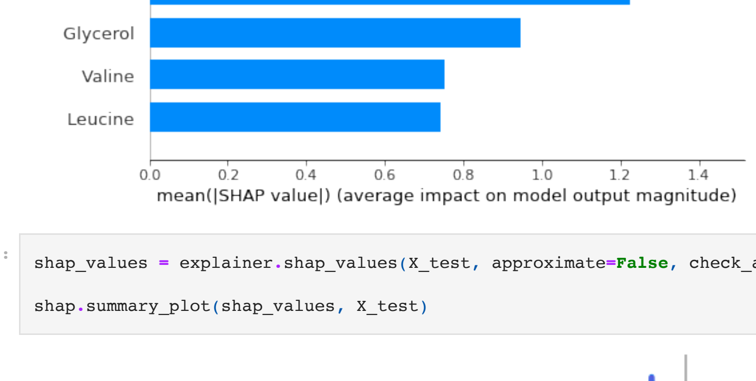

In [22]:
```python
shap_values = explainer.shap_values(X_test, approximate=False, check_additivity=False)

shap.summary_plot(shap_values, X_test)
```

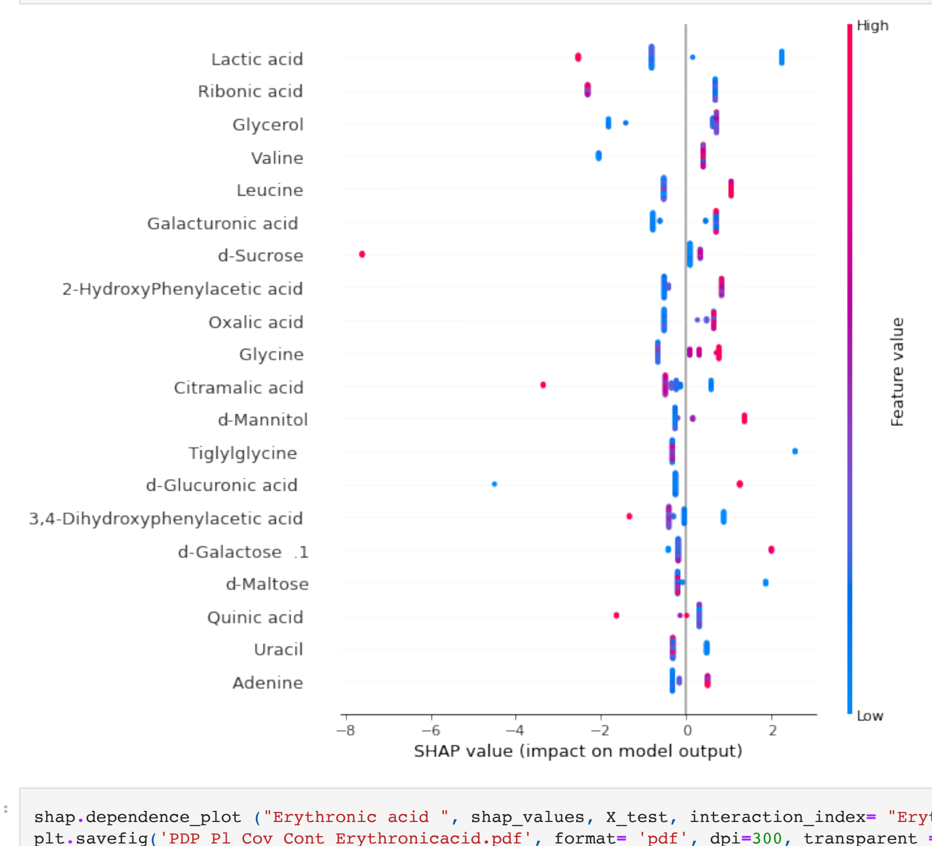

In [54]:
```python
shap.dependence_plot ("Erythronic acid ", shap_values, X_test, interaction_index= "Erythronic acid ", dot_size=20, x_jitter=3, alpha = 0.8, cmap=plt.get_cmap("cividis"
plt.savefig('PDP_Pl_Cov_Cont_Erythronicacid.pdf', format= 'pdf', dpi=300, transparent = True, bbox_inches='tight')
```

```
`should_run_async` will not call `transform_cell` automatically in the future. Please pass the result to `transformed_cell` argument and any exception that happen durin
g thetransform in `preprocessing_exc_tuple` in IPython 7.17 and above.
```

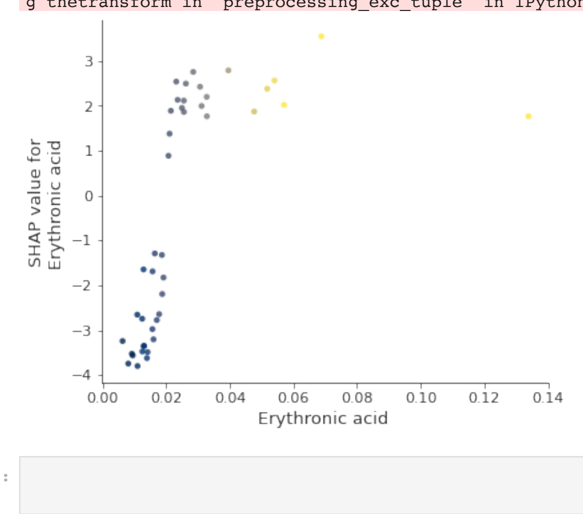

In [ ]:

Supplementary Table S3. Serum metabolites from healthy volunteers and COVID-19 positive patients

Metabolites (Molar %)	Control (n = 50)	Covid-19 (n = 126)	log_2FC (Covid-19 vs Control)	p-value	q-value
**Amino acid metabolism**					
**Alanine & aspartate metabolism**					
Alanine	5.1 (4.8 - 5.8)	4.6 (4.0 - 5.3)	-0.22	6.01E-05	2.26E-04
Glutamic acid	1.2 (9.4E-1 - 1.5)	2.0 (1.4 - 2.7)	0.83	1.00E-10	1.18E-09
**Arginine & proline metabolism**					
4-Hydroxyproline	1.6E-1 (1.2E-1 - 1.9E-1)	1.4E-1 (8.1E-2 - 2.0E-1)	-0.20	3.2E-02	6.5E-02
Ornithine	1.8 (1.6 - 2.2)	2.0 (1.5 - 2.4)	0.02	4.9E-01	6.0E-01
Oxoproline	7.0 (6.1 - 8.5)	7.8 (6.3 - 9.2)	0.10	8.3E-02	1.4E-01
Proline	3.3 (2.7 - 3.8)	2.7 (2.0 - 3.4)	-0.28	7.9E-04	2.3E-03
**Cysteine & methionine metabolism**					
5-formyl-THF	4.0E-4 (2.9E-4 - 5.6E-4)	1.2E-3 (6.9E-4 - 1.9E-3)	1.52	8.1E-13	1.5E-11
5-methyl-THF	3.7E-3 (2.6E-3 - 5.4E-3)	2.5E-3 (1.6E-3 - 4.2E-3)	-0.25	1.8E-03	5.0E-03
Betaine	54 (45 - 60)	40 (29 - 47)	-0.37	3.5E-07	2.0E-06
Homocysteine	11 (7.9 - 13)	14 (9.8 - 18)	0.26	5.6E-04	1.7E-03
Methionine	4.5E-1 (4.1E-1 - 5.1E-1)	3.9E-1 (3.1E-1 - 4.7E-1)	-0.24	4.0E-04	1.3E-03
SAH	4.8E-2 (4.1E-2 - 5.5E-2)	7.9E-2 (6.0E-2 - 1.3E-1)	1.09	8.4E-14	2.0E-12
SAM	1.5E-1 (1.1E-1 - 1.7E-1)	2.5E-1 (1.6E-1 - 3.6E-1)	1.19	1.5E-08	1.1E-07
**Glycine & serine metabolism**					
Dimethylglycine	3.3 (3.0 - 3.6)	3.1 (2.7 - 3.5)	-0.01	1.6E-01	2.5E-01
Glyceric acid	2.3 (2.0 - 2.6)	2.2 (1.8 - 2.5)	-0.92	6.8E-19	8.3E-17
Glycine	2.3E-1 (2.0E-1 - 2.6E-1)	1.1E-1 (9.0E-2 - 1.5E-1)	-0.12	5.5E-02	9.9E-02
Serine	2.4 (1.9 - 2.7)	1.8 (1.3 - 2.5)	-0.09	2.1E-01	3.0E-01
Threonine	5.8 (4.6 - 6.5)	4.7 (3.7 - 6.7)	-0.27	6.8E-04	2.1E-03
**Phenylalanine metabolism**					
Benzoic acid	3.8E-2 (3.2E-2 - 4.4E-2)	3.7E-2 (3.0E-2 - 4.6E-2)	-0.02	5.7E-01	6.6E-01
Hippuric acid	1.3E-1 (7.0E-2 - 2.3E-1)	3.3E-2 (1.4E-2 - 8.4E-2)	0.36	1.2E-06	6.7E-06
Hydrocinnamic acid	5.3E-3 (1.6E-3 - 8.8E-3)	9.1E-4 (2.7E-4 - 2.0E-3)	-2.39	2.2E-12	3.5E-11
Phenylalanine	1.2 (1.1 - 1.3)	1.5 (1.2 - 1.9)	-1.20	5.2E-10	4.7E-09
**Tryptophan metabolism**					
Indole-3-propanoic acid	4.0E-3 (2.6E-3 - 6.3E-3)	6.7E-4 (1.7E-4 - 1.6E-3)	-1.79	2.1E-15	9.5E-14
Indolelactic acid	1.2E-2 (9.1E-3 - 1.5E-2)	1.0E-2 (6.6E-3 - 1.3E-2)	0	1.6E-02	3.5E-02
**Tyrosine metabolism**					
Vanillylmandelic acid	1.0E-3 (6.4E-4 - 1.3E-3)	1.1E-3 (8.1E-4 - 1.7E-3)	0.55	1.9E-02	4.1E-02
**Valine, leucine & isoleucine metabolism**					
2-HydroxyButyric acid	5.0E-1 (4.3E-1 - 6.5E-1)	9.3E-1 (6.2E-1 - 1.3)	1.06	1.7E-01	2.6E-01
2-Hydroxyisobutyric acid	3.8E-2 (3.3E-2 - 4.4E-2)	6.4E-2 (4.8E-2 - 1.0E-1)	1	4.0E-09	3.3E-08
2-Hydroxyisovaleric acid	1.4E-1 (1.0E-1 - 1.7E-1)	2.1E-1 (1.3E-1 - 4.1E-1)	1.20	7.3E-06	3.3E-05
2-keto-3-methylvaleric acid	5.0E-2 (4.3E-2 - 5.5E-2)	3.2E-2 (2.6E-2 - 4.1E-2)	-0.63	4.4E-15	1.6E-13
3-hydroxybutyric acid/3-hydroxyisobutyric acid	3.4E-1 (2.6E-1 - 5.5E-1)	4.0E-1 (2.6E-1 - 1.0)	-0.41	7.4E-09	5.6E-08
3-Hydroxyisovaleric acid	3.7E-2 (2.8E-2 - 4.3E-2)	5.3E-2 (3.4E-2 - 8.9E-2)	0.94	5.2E-06	2.4E-05
3-methyl-2-oxobutyric acid	1.4E-2 (1.2E-2 - 1.6E-2)	1.1E-2 (8.4E-3 - 1.3E-2)	0.83	2.4E-10	2.4E-09
Isoleucine	1.2 (1.0 - 1.4)	1.1 (9.4E-1 - 1.3)	-0.13	9.8E-02	1.6E-01
Leucine	2.3 (2.1 - 2.6)	2.2 (1.8 - 2.5)	-0.14	2.1E-02	4.3E-02
Valine	3.8 (3.4 - 4.0)	3.3 (2.9 - 3.7)	-0.21	4.0E-05	1.6E-04
**Carbohydrate metabolism**					
**Ascorbate and aldrate metabolism**					

Saccharic acid	2.4E-2 (1.8E-2 - 3.1E-2)	5.3E-2 (3.3E-2 - 9.7E-2)	4.28	6.9E-13	1.3E-11
**C5-Branched dibasic acid metabolism**					
DL-2-Hydroxyglutaric acid	1.5E-2 (1.3E-2 - 1.8E-2)	2.1E-2 (1.7E-2 - 2.6E-2)	0.50	8.3E-09	6.2E-08
**Fructose & mannose metabolism**					
d-Fructose	1.4E-1 (1.3E-1 - 1.7E-1)	1.7E-1 (1.3E-1 - 2.6E-1)	0.91	5.4E-03	1.3E-02
d-Mannitol	1.4 (1.3 - 1.8)	9.8E-1 (2.8E-1 - 1.3)	-0.67	3.1E-07	1.8E-06
d-Mannonic acid	5.7E-1 (4.6E-1 - 7.1E-1)	3.4 (1.3 - 9.5)	3.99	2.7E-21	9.9E-19
**Galactose metabolism**					
d-Galactitol	9.4E-3 (6.3E-3 - 1.2E-2)	9.4E-3 (6.0E-3 - 1.6E-2)	0.55	6.6E-01	7.4E-01
**Glycolysis**					
Lactic acid	17 (16 - 19)	17 (14 - 19)	-0.09	1.3E-01	2.1E-01
3-Phosphoglyceric acid	1.6E-3 (1.2E-3 - 2.0E-3)	1.4E-3 (9.2E-4 - 2.0E-3)	-0.15	1.4E-01	2.2E-01
Glucose 6-phosphate	2.1E-3 (1.8E-3 - 2.3E-3)	2.6E-3 (2.1E-3 - 3.2E-3)	0.46	2.5E-06	1.3E-05
**Glyxolate and decarboxylate metabolism**					
Glycolic acid	9.0E-2 (8.3E-2 - 1.0E-1)	8.4E-2 (6.9E-2 - 1.0E-1)	-0.07	2.6E-02	5.4E-02
**Nucleotide sugar metabolism**					
d-Arabinose	2.1E-2 (1.8E-2 - 2.8E-2)	3.4E-2 (2.6E-2 - 4.7E-2)	1.04	3.1E-10	2.9E-09
d-Threitol	1.7E-2 (1.5E-2 - 1.9E-2)	2.5E-2 (2.0E-2 - 3.2E-2)	0.79	3.8E-13	7.7E-12
d-Xylose	1.7E-2 (1.5E-2 - 2.0E-2)	2.2E-2 (1.8E-2 - 3.6E-2)	0.78	1.2E-07	7.5E-07
Erythronic acid	1.3E-2 (1.2E-2 - 1.6E-2)	2.9E-2 (2.0E-2 - 5.2E-2)	1.60	4.8E-16	2.5E-14
Threonic acid	2.0E-1 (1.9E-1 - 2.4E-1)	3.8E-1 (2.9E-1 - 5.9E-1)	1.25	3.0E-17	1.8E-15
Xylonic acid	2.7E-2 (2.3E-2 - 3.1E-2)	4.2E-2 (3.0E-2 - 6.1E-2)	0.90	1.7E-10	1.9E-09
**Pentose glucoronate interconversion metabolism**					
d-Arabitol	5.0E-3 (4.3E-3 - 6.6E-3)	9.0E-3 (7.1E-3 - 1.4E-2)	1.13	3.4E-14	9.6E-13
d-Xylitol	9.7E-4 (8.4E-4 - 1.1E-3)	2.0E-3 (1.5E-3 - 4.5E-3)	2.21	9.0E-20	1.6E-17
Galactonic acid	1.5E-2 (5.0E-3 - 1.8E-2)	2.7E-2 (1.2E-2 - 4.5E-2)	4.02	4.3E-05	1.7E-04
Galacturonic acid	2.7E-2 (2.3E-2 - 3.1E-2)	8.4E-2 (4.8E-2 - 1.7E-1)	2.66	1.4E-17	1.0E-15
myo-Inositol	3.4E-1 (2.9E-1 - 3.8E-1)	4.1E-1 (2.9E-1 - 5.8E-1)	0.41	7.1E-03	1.7E-02
**Pentose phosphate metabolism**					
Sedoheptulose	3.3E-2 (2.6E-2 – 4.2E-2)	4.8E-2 (3.6E-2 – 6.4E-2)	0.57	1.1E-07	6.4E-07
**Sucrose metabolism**					
d-Sucrose	6.0E-3 (4.1E-3 - 7.7E-3)	8.1E-3 (5.4E-3 - 2.0E-2)	1.77	3.5E-04	1.2E-03
Maltose	6.1E-3 (5.3E-3 - 7.0E-3)	1.4E-2 (1.0E-2 - 2.2E-2)	1.78	1.7E-18	1.5E-16
**Tricarboxylic acid cycle metabolism**					
alpha-ketoglutaric acid	3.9E-2 (3.2E-2 - 4.5E-2)	7.3E-2 (5.4E-2 - 1.0E-1)	1.07	1.4E-13	3.2E-12
Citric acid	2.0 (1.6 - 2.4)	1.8 (1.2 - 2.7)	-0.04	1.4E-01	2.2E-01
Fumaric acid	9.6E-3 (7.9E-3 - 1.1E-2)	1.0E-2 (7.6E-3 - 1.3E-2)	0.15	2.3E-01	3.2E-01
Glutamine	14 (12E - 16)	9.7 (5.8 - 14)	-0.52	8.5E-08	5.3E-07
Malic acid	3.1E-2 (2.8E-2 - 3.6E-2)	4.1E-2 (3.1E-2 - 5.4E-2)	0.48	2.8E-06	1.4E-05
Pyruvic acid	7.5E-1 (5.4E-1 - 1.1)	9.2E-1 (6.5E-1 - 1.3)	0.30	1.7E-02	3.5E-02
Succinic acid	8.4E-2 (7.5E-2 - 9.9E-2)	7.6E-2 (6.0E-2 - 9.1E-2)	-0.14	1.3E-02	2.8E-02
**Energy metabolism**					
**Methane metabolism**					
TMAO	7.1 (5.1 - 14)	18 (7.8 - 30)	0.63	2.9E-04	9.7E-04
**Oxidative phosphorylation**					
Phosphoric acid	5.9 (5.1 - 6.8)	4.2 (3.5 - 5.4)	-0.46	5.3E-09	4.1E-08
**Lipid metabolism**					
**Glycerolipid metabolism**					
Choline	18 (16 - 21)	19 (16 - 25)	0.11	2.4E-01	3.3E-01
Ethanolamine	9.5E-2 (9.0E-2 - 1.0E-1)	8.6E-2 (6.9E-2 - 1.1E-1)	-0.09	3.2E-03	8.1E-03
Glycerol	1.2 (9.7E-1 - 1.8)	8.0E-1 (5.5E-1 - 1.2)	-0.50	9.0E-08	5.5E-07
Glycerol-1-phosphate	3.4E-2 (2.7E-2 - 4.1E-2)	2.7E-2 (2.0E-2 - 3.5E-2)	-0.27	2.9E-04	9.7E-04
**Lipids**					
Dodecanoic acid	8.3E-2 (6.5E-2 - 1.1E-1)	6.3E-2 (4.8E-2 - 8.0E-2)	-0.69	4.0E-06	1.9E-05

Linoleic acid	4.6E-1 (3.0E-1 - 6.1E-1)	3.4E-1 (1.9E-1 - 6.1E-1)	-0.13	6.1E-02	1.1E-01
Oleic acid	2.5 (1.5 - 3.6)	3.1 (1.8 - 4.7)	0.32	5.6E-02	1.0E-01
Tetradecanoic acid	2.6E-1 (2.3E-1 - 3.2E-1)	2.4E-1 (1.9E-1 - 3.1E-1)	-0.23	6.4E-02	1.1E-01
**Primary bile acid biosynthesis**					
Taurine	3.1 (2.6 - 4.3)	2.7 (1.7 - 4.4)	-0.20	8.5E-02	1.4E-01
**Metabolism of cofactors**					
**Cofactor biosynthesis**					
alpha-tocopherol	3.6E-2 (3.0E-2 - 5.6E-2)	1.4E-2 (7.4E-3 - 2.4E-2)	-1.19	1.8E-12	3.2E-11
**Nucleotide metabolism**					
**Purine & pirymidine**					
Ethylmalonic acid	1.1 (9.5E-1 - 1.5)	7.5E-1 (5.2E-1 - 9.7E-1)	-0.55	2.2E-10	2.2E-09
Hypoxanthine	4.0E-2 (2.8E-2 - 4.7E-2)	4.9E-2 (3.1E-2 - 8.4E-2)	0.70	9.0E-03	2.1E-02
Ribonic acid	7.6E-3 (6.1E-3 - 8.9E-3)	1.3E-2 (7.6E-3 - 2.1E-2)	1.03	2.8E-06	1.4E-05
Uracil	1.6E-3 (1.3E-3 - 2.0E-3)	2.2E-3 (1.7E-3 - 2.8E-3)	0.59	3.6E-06	1.7E-05
Uric acid	6.6 (5.2 - 9.8)	4.7 (2.8 - 7.6)	-0.51	7.7E-05	2.8E-04
**Xenobiotic metabolism**					
**Benzoate degradation**					
4-Hydroxybenzoic acid	2.1E-2 (2.4E-3 - 2.3E-2)	1.7E-2 (1.2E-2 - 2.1E-2)	0.13	6.9E-01	7.6E-01

Results are shown as the molar percentage of total metabolites. Median ± interquartile range (25 – 75) was calculated, and statistical significance was assessed with Wilcoxon rank-sum test and false-discovery rate (FDR q<0.05) correction by the Benjamini-Hochberg method. SAH, s-adenosylhomocisteine; SAM, s-adenosylmethionine; THF, tetrahydrofolate; TMAO, trymethylamine N-oxide.

Supplementary Table S4. Serum metabolites from healthy volunteers and COVID-19 negative patients

Metabolites (Molar %)	Control (n = 50)	No-Covid-19 (n = 45)	log_2FC (No-Covid-19 vs Control)	p-value	q-value
**Amino acid metabolism**					
**Alanine & aspartate metabolism**					
Alanine	5.1 (4.8 - 5.8)	5.6 (5.0 - 6.1)	0.09	4.2E-02	8.09E-02
Glutamic acid	1.2 (9.4E-1 - 1.5)	2.1 (1.7 - 2.6)	0.93	2.8E-10	2.68E-09
**Arginine & proline metabolism**					
4-Hydroxyproline	1.6E-1 (1.2E-1 - 1.9E-1)	1.6E-1 (1.2E-1 - 2.1E-1)	-0.08	9.9E-01	9.91E-01
Ornithine	1.8 (1.6 - 2.2)	2.1 (1.6 - 2.7)	0.14	2.3E-01	3.20E-01
Oxoproline	7.0 (6.1 - 8.5)	7.5 (6.7 - 8.6)	0.07	2.3E-01	3.28E-01
Proline	3.3 (2.7 - 3.8)	3.3 (2.8 - 4.0)	0.09	4.8E-01	5.90E-01
**Cysteine & methionine metabolism**					
5-formyl-THF	4.0E-4 (2.9E-4 - 5.6E-4)	6.4E-4 (4.5E-4 - 1.0E-3)	0.59	4.7E-05	1.82E-04
5-methyl-THF	3.7E-3 (2.6E-3 - 5.4E-3)	2.2E-3 (1.0E-3 - 3.4E-3)	-0.67	2.3E-04	7.95E-04
Betaine	54 (45 - 60)	40 (28 - 51)	-0.32	3.9E-04	1.27E-03
Homocysteine	11 (7.9 - 13)	14 (11 - 18)	0.34	8.6E-04	2.50E-03
Methionine	4.5E-1 (4.1E-1 - 5.1E-1)	4.1E-1 (3.4E-1 - 5.0E-1)	-0.12	4.4E-02	8.26E-02
SAH	4.8E-2 (4.1E-2 - 5.5E-2)	5.4E-2 (4.3E-2 - 7.4E-2)	0.35	4.7E-02	8.73E-02
SAM	1.5E-1 (1.1E-1 - 1.7E-1)	1.6E-1 (1.3E-1 - 2.5E-1)	0.43	3.6E-02	7.17E-02
**Glycine & serine metabolism**					
Dimethylglycine	5.8 (4.6 - 6.5)	4.9 (3.9 - 6.5)	-0.24	1.6E-03	4.48E-03
Glyceric acid	2.3E-1 (2.0E-1 - 2.6E-1)	1.5E-1 (1.0E-1 - 1.9E-1)	-0.01	7.5E-01	8.13E-01
Glycine	3.3 (3.0 - 3.6)	3.3 (2.9 - 3.6)	-0.08	2.6E-01	3.53E-01
Serine	2.3 (2.0 - 2.6)	2.3 (2.1 - 2.7)	-0.58	1.8E-08	1.28E-07
Threonine	2.4 (1.9 - 2.7)	1.8 (1.7 - 2.3)	0.04	4.0E-01	5.13E-01
**Phenylalanine metabolism**					
Benzoic acid	3.8E-2 (3.2E-2 - 4.4E-2)	3.9E-2 (3.0E-2 - 5.3E-2)	0.09	8.1E-01	8.62E-01
Hippuric acid	1.3E-1 (7.0E-2 - 2.3E-1)	9.4E-2 (4.4E-2 - 1.9E-1)	0.32	5.7E-06	2.61E-05
hydrocinnamic acid	5.3E-3 (1.6E-3 - 8.8E-3)	1.2E-3 (3.8E-4 - 2.2E-3)	-1.86	1.3E-06	7.02E-06
Phenylalanine	1.2 (1.1 - 1.3)	1.5 (1.2 - 1.7)	-0.08	8.7E-02	1.46E-01
**Tryptophan metabolism**					
Indole-3-propanoic acid	4.0E-3 (2.6E-3 - 6.3E-3)	1.7E-3 (3.2E-4 - 3.3E-3)	-1.17	1.4E-06	7.34E-06
Indolelactic acid	1.2E-2 (9.1E-3 - 1.5E-2)	1.1E-2 (7.0E-3 - 1.5E-2)	0.02	4.2E-01	5.29E-01
**Tyrosine metabolism**					
Vanillylmandelic acid	1.0E-3 (6.4E-4 - 1.3E-3)	1.7E-3 (1.3E-3 - 2.3E-3)	0.90	1.3E-06	7.18E-06
**Valine, leucine & isoleucine metabolism**					
2-HydroxyButyric acid	5.0E-1 (4.3E-1 - 6.5E-1)	7.1E-1 (5.4E-1 - 1.0)	0.48	2.1E-01	3.00E-01
2-Hydroxyisobutyric acid	3.8E-2 (3.3E-2 - 4.4E-2)	5.7E-2 (5.0E-2 - 1.3E-1)	0.55	1.1E-03	3.24E-03
2-Hydroxyisovaleric acid	1.4E-1 (1.0E-1 - 1.7E-1)	1.8E-1 (1.2E-1 - 2.7E-1)	0.66	2.6E-03	6.85E-03
2-keto-3-methylvaleric acid	5.0E-2 (4.3E-2 - 5.5E-2)	3.2E-2 (2.3E-2 - 4.3E-2)	-0.64	8.4E-10	7.42E-09
3-hydroxybutyric acid/3-hydroxyisobutyric acid	3.4E-1 (2.6E-1 - 5.5E-1)	4.3E-1 (2.9E-1 - 7.1E-1)	-0.52	6.9E-08	4.43E-07
3-Hydroxyisovaleric acid	3.7E-2 (2.8E-2 - 4.3E-2)	3.9E-2 (3.2E-2 - 5.5E-2)	0.29	1.2E-01	1.95E-01
3-methyl-2-oxobutyric acid	1.4E-2 (1.2E-2 - 1.6E-2)	1.0E-2 (7.2E-3 - 1.3E-2)	1.09	3.7E-08	2.50E-07
Isoleucine	1.2 (1.0 - 1.4)	1.1 (9.4E-1 - 1.3)	-0.07	3.1E-01	4.07E-01
Leucine	2.3 (2.1 - 2.6)	2.1 (1.8 - 2.4)	-0.13	6.4E-03	1.54E-02
Valine	3.8 (3.4 - 4.0)	3.3 (2.9 - 3.7)	-0.16	4.2E-04	1.33E-03
**Carbohydrate metabolism**					
**Ascorbate and aldrate metabolism**					

Saccharic acid	2.4E-2 (1.8E-2 - 3.1E-2)	5.1E-2 (3.9E-2 - 7.8E-2)	1.33	2.6E-11	3.51E-10
**C5-Branched dibasic acid metabolsim**					
DL-2-Hydroxyglutaric acid	1.5E-2 (1.3E-2 - 1.8E-2)	2.1E-2 (1.7E-2 - 2.4E-2)	0.46	2.9E-07	1.69E-06
**Fructose & mannose metabolism**					
d-Fructose	1.4E-1 (1.3E-1 - 1.7E-1)	2.1E-1 (1.3E-1 - 3.8E-1)	0.70	1.0E-03	2.86E-03
d-Mannitol	1.4 (1.3 - 1.8)	1.2 (1.6E-1 - 2.4)	-0.42	4.6E-03	1.14E-02
d-Mannonic acid	5.7E-1 (4.6E-1 - 7.1E-1)	1.8 (1.1 - 2.8)	2.24	3.0E-14	9.06E-13
**Galactose metabolism**					
d-Galactitol	9.4E-3 (6.3E-3 - 1.2E-2)	1.2E-2 (7.5E-3 - 1.6E-2)	0.22	1.4E-01	2.22E-01
**Glycolysis**					
3-Phosphoglyceric acid	1.6E-3 (1.2E-3 - 2.0E-3)	1.8E-3 (1.2E-3 - 2.4E-3)	0.18	2.0E-01	2.88E-01
Glucose 6-phosphate	2.1E-3 (1.8E-3 - 2.3E-3)	2.1E-3 (1.6E-3 - 2.9E-3)	0.17	8.7E-01	9.01E-01
Lactic acid	17 (16 - 19)	19 (17 - 22)	0.15	2.4E-03	6.41E-03
**Glyxolate and decarboxylate metabolism**					
Glycolic acid	9.0E-2 (8.3E-2 - 1.0E-1)	8.5E-2 (7.1E-2 - 9.5E-2)	-0.16	3.3E-02	6.54E-02
**Nucleotide sugar metabolism**					
d-Arabinose	2.1E-2 (1.8E-2 - 2.8E-2)	4.1E-2 (3.1E-2 - 6.6E-2)	1.08	1.7E-10	1.90E-09
d-Threitol	1.7E-2 (1.5E-2 - 1.9E-2)	3.3E-2 (2.5E-2 - 3.9E-2)	1.18	7.9E-14	2.04E-12
d-Xylose	1.7E-2 (1.5E-2 - 2.0E-2)	3.0E-2 (2.2E-2 - 3.7E-2)	0.96	4.8E-09	3.78E-08
Erythronic acid	1.3E-2 (1.2E-2 - 1.6E-2)	3.1E-2 (2.2E-2 - 4.5E-2)	1.49	1.9E-12	3.18E-11
Threonic acid	2.0E-1 (1.9E-1 - 2.4E-1)	4.4E-1 (3.2E-1 - 5.5E-1)	1.31	2.3E-13	4.91E-12
Xylonic acid	2.7E-2 (2.3E-2 - 3.1E-2)	4.8E-2 (3.7E-2 - 6.4E-2)	1.01	7.5E-11	9.39E-10
**Pentose glucoronate interconversion metabolism**					
d-Arabitol	5.0E-3 (4.3E-3 - 6.6E-3)	9.9E-3 (8.4E-3 - 1.4E-2)	1.21	8.4E-12	1.18E-10
d-Xylitol	9.7E-4 (8.4E-4 - 1.1E-3)	1.4E-3 (1.0E-3 - 2.0E-3)	0.83	5.0E-07	2.78E-06
Galactonic acid	1.5E-2 (5.0E-3 - 1.8E-2)	2.7E-2 (1.1E-2 - 4.8E-2)	1.46	7.0E-05	2.59E-04
Galacturonic acid	2.7E-2 (2.3E-2 - 3.1E-2)	6.0E-2 (3.9E-2 - 9.0E-2)	1.41	1.0E-10	1.18E-09
myo-Inositol	3.4E-1 (2.9E-1 - 3.8E-1)	5.0E-1 (3.8E-1 - 6.0E-1)	0.72	3.5E-08	2.44E-07
**Pentose phosphate metabolism**					
Sedoheptulose	3.3E-2 (2.6E-2 – 4.2E-2)	6.5E-2 (4.3E-2 - 1.0E-1)	1.14	1.8E-09	1.56E-08
**Sucrose metabolism**					
d-Sucrose	6.0E-3 (4.1E-3 - 7.7E-3)	1.6E-2 (1.0E-2 - 3.4E-2)	3.01	6.2E-12	9.34E-11
Maltose	6.1E-3 (5.3E-3 - 7.0E-3)	1.2E-2 (9.8E-3 - 1.8E-2)	1.31	6.8E-12	9.96E-11
**Tricarboxylic acid cycle metabolism**					
alpha-ketoglutaric acid	3.9E-2 (3.2E-2 - 4.5E-2)	5.9E-2 (4.7E-2 - 9.5E-2)	1.07	2.8E-06	1.38E-05
Citric acid	2.0 (1.6 - 2.4)	2.4 (1.8 - 3.6)	0.37	1.4E-02	3.06E-02
Fumaric acid	9.6E-3 (7.9E-3 - 1.1E-2)	1.4E-2 (1.1E-2 - 1.7E-2)	0.69	2.9E-08	2.01E-07
Glutamine	14 (12E - 16)	11 (7.4 - 14)	-0.41	4.9E-04	1.53E-03
Malic acid	3.1E-2 (2.8E-2 - 3.6E-2)	5.6E-2 (4.5E-2 - 7.9E-2)	1.10	3.2E-11	4.15E-10
Pyruvic acid	7.5E-1 (5.4E-1 - 1.1)	6.9E-1 (4.8E-1 - 1.0)	-0.03	6.0E-01	6.92E-01
Succinic acid	8.4E-2 (7.5E-2 - 9.9E-2)	1.3E-1 (1.1E-1 - 1.6E-1)	0.65	2.0E-10	2.08E-09
**Energy metabolism**					
**Methane metabolism**					
TMAO	7.1 (5.1 - 14)	16 (7.7 - 27)	0.56	6.8E-03	1.60E-02
**Oxidative phosphorylation**					
Phosphoric acid	5.9 (5.1 - 6.8)	2.6 (2.1 - 3.4)	-1.11	7.8E-15	2.59E-13
**Lipid metabolism**					
**Glycerolipid metabolism**					
Choline	18 (16 - 21)	18 (16 - 21)	0.05	7.7E-01	8.21E-01
Ethanolamine	9.5E-2 (9.0E-2 - 1.0E-1)	1.0E-1 (8.7E-2 - 1.3E-1)	0.24	4.5E-02	8.36E-02
Glycerol	1.2 (9.7E-1 - 1.8)	9.0E-1 (6.8E-1 - 1.4)	-0.26	4.1E-03	1.03E-02
Glycerol-1-phosphate	3.4E-2 (2.7E-2 - 4.1E-2)	3.4E-2 (2.7E-2 - 4.3E-2)	0.12	7.6E-01	8.15E-01
**Lipids**					
Dodecanoic acid	8.3E-2 (6.5E-2 - 1.1E-1)	6.1E-2 (4.8E-2 - 7.3E-2)	-0.70	5.6E-05	2.15E-04
Linoleic acid	4.6E-1 (3.0E-1 - 6.1E-1)	2.9E-1 (1.6E-1 - 4.4E-1)	-0.59	7.2E-04	2.16E-03

Oleic acid	2.5 (1.5 - 3.6)	2.0 (1.1 - 4.1)	-0.01	6.7E-01	7.46E-01
Tetradecanoic acid	2.6E-1 (2.3E-1 - 3.2E-1)	2.2E-1 (1.7E-1 - 2.7E-1)	-0.37	1.3E-03	3.76E-03
**Primary bile acid biosynthesis**					
Taurine	3.1 (2.6 - 4.3)	3.3 (2.5 - 4.9)	0.02	7.5E-01	8.10E-01
**Metabolism of cofactors**					
**Cofactor biosynthesis**					
alpha-tocopherol	3.6E-2 (3.0E-2 - 5.6E-2)	1.1E-2 (5.9E-3 - 2.0E-2)	-1.43	1.8E-10	1.94E-09
**Nucleotide metabolism**					
**Purine & pyrimidine**					
Ethylmalonic acid	1.1 (9.5E-1 - 1.5)	7.7E-1 (6.3E-1 - 1.1)	-0.42	2.6E-05	1.07E-04
Hypoxanthine	4.0E-2 (2.8E-2 - 4.7E-2)	1.2E-1 (4.6E-2 - 2.7E-1)	2.11	6.9E-08	4.43E-07
Ribonic acid	7.6E-3 (6.1E-3 - 8.9E-3)	1.4E-2 (1.1E-2 - 2.4E-2)	1.25	9.6E-10	8.33E-09
Uracil	1.6E-3 (1.3E-3 - 2.0E-3)	1.7E-3 (1.2E-3 - 2.0E-3)	0.09	1.0E+00	9.99E-01
Uric acid	6.6 (5.2 - 9.8)	7.5 (5.2 - 9.6)	-0.02	9.1E-01	9.26E-01
**Xenobiotic metabolism**					
**Benzoate degradation**					
4-Hydroxybenzoic acid	2.1E-2 (2.4E-3 - 2.3E-2)	2.2E-2 (1.8E-2 - 2.7E-2)	0.78	1.3E-02	2.78E-02

Results are shown as the molar percentage of total metabolites. Median ± interquartile range (25 – 75) was calculated, and statistical significance was assessed with Wilcoxon rank-sum test and false-discovery rate (FDR q<0.05) correction by the Benjamini-Hochberg method. SAH, s-adenosylhomocisteine; SAM, s-adenosylmethionine; THF, tetrahydrofolate; TMAO, trymethylamine N-oxide.

Supplementary Table S5. Serum metabolites from COVID-19 positive and negative patients

Metabolites (Molar %)	Covid-19 (n = 126)	No-Covid-19 (n = 45)	log_2FC (Covid-19 vs No-Covid-19)	p-value	q-value
**Amino acid metabolism**					
**Alanine & aspartate metabolism**					
Alanine	4.6 (4.0 - 5.3)	5.6 (5.0 - 6.1)	-0.31	4.4E-08	2.9E-07
Glutamic acid	2.0 (1.4 - 2.7)	2.1 (1.7 - 2.6)	-0.10	4.6E-01	5.7E-01
**Arginine & proline metabolism**					
4-Hydroxyproline	1.4E-1 (8.1E-2 - 2.0E-1)	1.6E-1 (1.2E-1 - 2.1E-1)	-0.11	5.4E-02	9.8E-02
Ornithine	2.0 (1.5 - 2.4)	2.1 (1.6 - 2.7)	-0.12	4.1E-01	5.2E-01
Oxoproline	7.8 (6.3 - 9.2)	7.5 (6.7 - 8.6)	0.03	6.3E-01	7.1E-01
Proline	2.7 (2.0 - 3.4)	3.3 (2.8 - 4.0)	-0.37	1.1E-04	3.7E-04
**Cysteine & methionine metabolism**					
5-formyl-THF	1.2E-3 (6.9E-4 - 1.9E-3)	6.4E-4 (4.5E-4 - 1.0E-3)	0.93	4.4E-05	1.8E-04
5-methyl-THF	2.5E-3 (1.6E-3 - 4.2E-3)	2.2E-3 (1.0E-3 - 3.4E-3)	0.42	1.4E-01	2.2E-01
Betaine	40 (29 - 47)	40 (28 - 51)	-0.05	6.4E-01	7.2E-01
Homocysteine	14 (9.8 - 18)	14 (11 - 18)	-0.09	3.9E-01	5.1E-01
Methionine	3.9E-1 (3.1E-1 - 4.7E-1)	4.1E-1 (3.4E-1 - 5.0E-1)	-0.12	2.3E-01	3.2E-01
SAH	7.9E-2 (6.0E-2 - 1.3E-1)	5.4E-2 (4.3E-2 - 7.4E-2)	0.74	4.7E-06	2.2E-05
SAM	2.5E-1 (1.6E-1 - 3.6E-1)	1.6E-1 (1.3E-1 - 2.5E-1)	0.77	1.9E-03	5.2E-03
**Glycine & serine metabolism**					
Dimethylglycine	3.1 (2.7 - 3.5)	4.9 (3.9 - 6.5)	-0.03	5.0E-01	6.1E-01
Glyceric acid	2.2 (1.8 - 2.5)	1.5E-1 (1.0E-1 - 1.9E-1)	-0.11	5.0E-02	9.1E-02
Glycine	1.1E-1 (9.0E-2 - 1.5E-1)	3.3 (2.9 - 3.6)	0.07	9.2E-01	9.4E-01
Serine	1.8 (1.3 - 2.5)	2.3 (2.1 - 2.7)	-0.34	1.4E-03	3.8E-03
Threonine	4.7 (3.7 - 6.7)	1.8 (1.7 - 2.3)	-0.13	2.5E-02	5.2E-02
**Phenylalanine metabolism**					
Benzoic acid	3.7E-2 (3.0E-2 - 4.6E-2)	3.9E-2 (3.0E-2 - 5.3E-2)	-0.11	4.6E-01	5.8E-01
Hippuric acid	3.3E-2 (1.4E-2 - 8.4E-2)	9.4E-2 (4.4E-2 - 1.9E-1)	0.04	7.8E-01	8.3E-01
Hydrocinnamic acid	9.1E-4 (2.7E-4 - 2.0E-3)	1.2E-3 (3.8E-4 - 2.2E-3)	-0.53	2.0E-01	3.0E-01
Phenylalanine	1.5 (1.2 - 1.9)	1.5 (1.2 - 1.7)	-1.12	2.1E-05	8.6E-05
**Tryptophan metabolism**					
Indole-3-propanoic acid	6.7E-4 (1.7E-4 - 1.6E-3)	1.7E-3 (3.2E-4 - 3.3E-3)	-0.62	6.1E-03	1.5E-02
Indolelactic acid	1.0E-2 (6.6E-3 - 1.3E-2)	1.1E-2 (7.0E-3 - 1.5E-2)	-0.02	4.0E-01	5.1E-01
**Tyrosine metabolism**					
Vanillylmandelic acid	1.1E-3 (8.1E-4 - 1.7E-3)	1.7E-3 (1.3E-3 - 2.3E-3)	-0.35	1.9E-03	5.2E-03
**Valine, leucine & isoleucine metabolism**					
2-HydroxyButyric acid	9.3E-1 (6.2E-1 - 1.3)	7.1E-1 (5.4E-1 - 1.0)	0.59	9.1E-01	9.3E-01
2-Hydroxyisobutyric acid	6.4E-2 (4.8E-2 - 1.0E-1)	5.7E-2 (5.0E-2 - 1.3E-1)	0.45	2.0E-02	4.1E-02
2-Hydroxyisovaleric acid	2.1E-1 (1.3E-1 - 4.1E-1)	1.8E-1 (1.2E-1 - 2.7E-1)	0.54	1.9E-01	2.8E-01
2-keto-3-methylvaleric acid	3.2E-2 (2.6E-2 - 4.1E-2)	3.2E-2 (2.3E-2 - 4.3E-2)	0.01	8.5E-01	8.9E-01
3-hydroxybutyric acid/3-hydroxyisobutyric acid	4.0E-1 (2.6E-1 - 1.0)	4.3E-1 (2.9E-1 - 7.1E-1)	0.11	1.9E-01	2.8E-01
3-Hydroxyisovaleric acid	5.3E-2 (3.4E-2 - 8.9E-2)	3.9E-2 (3.2E-2 - 5.5E-2)	0.65	5.8E-03	1.4E-02
3-methyl-2-oxobutyric acid	1.1E-2 (8.4E-3 - 1.3E-2)	1.0E-2 (7.2E-3 - 1.3E-2)	-0.26	9.7E-01	9.8E-01
Isoleucine	1.1 (9.4E-1 - 1.3)	1.1 (9.4E-1 - 1.3)	-0.05	6.0E-01	6.9E-01
Leucine	2.2 (1.8 - 2.5)	2.1 (1.8 - 2.4)	-0.01	7.0E-01	7.8E-01
Valine	3.3 (2.9 - 3.7)	3.3 (2.9 - 3.7)	-0.05	8.3E-01	8.8E-01
**Carbohydrate metabolism**					

**Ascorbate and aldrate metabolism**					
Saccharic acid	5.3E-2 (3.3E-2 - 9.7E-2)	5.1E-2 (3.9E-2 - 7.8E-2)	2.95	5.2E-01	6.3E-01
**C5-Branched dibasic acid metabolism**					
DL-2-Hydroxyglutaric acid	2.1E-2 (1.7E-2 - 2.6E-2)	2.1E-2 (1.7E-2 - 2.4E-2)	0.04	6.5E-01	7.3E-01
**Fructose & mannose metabolism**					
d-Fructose	1.7E-1 (1.3E-1 - 2.6E-1)	2.1E-1 (1.3E-1 - 3.8E-1)	0.21	2.8E-01	3.8E-01
d-Mannitol	9.8E-1 (2.8E-1 - 1.3)	1.2 (1.6E-1 - 2.4)	-0.25	5.7E-01	6.6E-01
d-Mannonic acid	3.4 (1.3 - 9.5)	1.8 (1.1 - 2.8)	1.74	2.1E-03	5.5E-03
**Galactose metabolism**					
d-Galactitol	9.4E-3 (6.0E-3 - 1.6E-2)	1.2E-2 (7.5E-3 - 1.6E-2)	0.33	3.7E-01	4.8E-01
**Glycolisis**					
3-Phosphoglyceric acid	17 (14 - 19)	1.8E-3 (1.2E-3 - 2.4E-3)	-0.33	5.0E-03	1.2E-02
Glucose 6-phosphate	1.4E-3 (9.2E-4 - 2.0E-3)	2.1E-3 (1.6E-3 - 2.9E-3)	0.29	3.5E-03	8.9E-03
Lactic acid	2.6E-3 (2.1E-3 - 3.2E-3)	19 (17 - 22)	-0.23	2.0E-05	8.4E-05
**Glyxolate and decarboxylate metabolism**					
Glycolic acid	8.4E-2 (6.9E-2 - 1.0E-1)	8.5E-2 (7.1E-2 - 9.5E-2)	0.09	7.3E-01	7.9E-01
**Nucleotide sugar metabolism**					
d-Arabinose	3.4E-2 (2.6E-2 - 4.7E-2)	4.1E-2 (3.1E-2 - 6.6E-2)	-0.04	8.1E-03	1.9E-02
d-Threitol	2.5E-2 (2.0E-2 - 3.2E-2)	3.3E-2 (2.5E-2 - 3.9E-2)	-0.39	2.8E-03	7.3E-03
d-Xylose	2.2E-2 (1.8E-2 - 3.6E-2)	3.0E-2 (2.2E-2 - 3.7E-2)	-0.17	4.0E-02	7.8E-02
Erythronic acid	2.9E-2 (2.0E-2 - 5.2E-2)	3.1E-2 (2.2E-2 - 4.5E-2)	0.11	8.8E-01	9.1E-01
Threonic acid	3.8E-1 (2.9E-1 - 5.9E-1)	4.4E-1 (3.2E-1 - 5.5E-1)	-0.06	4.7E-01	5.8E-01
Xylonic acid	4.2E-2 (3.0E-2 - 6.1E-2)	4.8E-2 (3.7E-2 - 6.4E-2)	-0.10	1.6E-01	2.5E-01
**Pentose glucoronate interconversion metabolism**					
d-Arabitol	9.0E-3 (7.1E-3 - 1.4E-2)	9.9E-3 (8.4E-3 - 1.4E-2)	-0.08	2.2E-01	3.1E-01
d-Xylitol	2.0E-3 (1.5E-3 - 4.5E-3)	1.4E-3 (1.0E-3 - 2.0E-3)	1.38	5.5E-05	2.1E-04
Galactonic acid	2.7E-2 (1.2E-2 - 4.5E-2)	2.7E-2 (1.1E-2 - 4.8E-2)	2.56	7.2E-01	7.9E-01
Galacturonic acid	8.4E-2 (4.8E-2 - 1.7E-1)	6.0E-2 (3.9E-2 - 9.0E-2)	1.25	5.6E-03	1.4E-02
myo-Inositol	4.1E-1 (2.9E-1 - 5.8E-1)	5.0E-1 (3.8E-1 - 6.0E-1)	-0.32	9.6E-03	2.2E-02
**Pentose phosphate metabolism**					
Sedoheptulose	4.8E-2 (3.6E-2 – 6.4E-2)	6.5E-2 (4.3E-2 - 1.0E-1)	-0.56	1.5E-03	4.1E-03
**Sucrose metabolism**					
d-Sucrose	8.1E-3 (5.4E-3 - 2.0E-2)	1.6E-2 (1.0E-2 - 3.4E-2)	-1.24	1.1E-05	4.9E-05
Maltose	1.4E-2 (1.0E-2 - 2.2E-2)	1.2E-2 (9.8E-3 - 1.8E-2)	0.48	2.4E-01	3.3E-01
**Tricarboxylic acid cycle metabolism**					
alpha-ketoglutaric acid	7.3E-2 (5.4E-2 - 1.0E-1)	5.9E-2 (4.7E-2 - 9.5E-2)	-0.01	7.2E-02	1.3E-01
Citric acid	1.8 (1.2 - 2.7)	2.4 (1.8 - 3.6)	-0.40	7.1E-04	2.2E-03
Fumaric acid	1.0E-2 (7.6E-3 - 1.3E-2)	1.4E-2 (1.1E-2 - 1.7E-2)	-0.53	5.3E-06	2.4E-05
Glutamine	9.7 (5.8 - 14)	11 (7.4 - 14)	-0.12	2.4E-01	3.3E-01
Malic acid	4.1E-2 (3.1E-2 - 5.4E-2)	5.6E-2 (4.5E-2 - 7.9E-2)	-0.61	1.0E-05	4.4E-05
Pyruvic acid	9.2E-1 (6.5E-1 - 1.3)	6.9E-1 (4.8E-1 - 1.0)	0.33	8.4E-03	1.9E-02
Succinic acid	7.6E-2 (6.0E-2 - 9.1E-2)	1.3E-1 (1.1E-1 - 1.6E-1)	-0.79	4.4E-15	1.6E-13
**Energy metabolism**					
**Methane metabolism**					
TMAO	18 (7.8 - 30)	16 (7.7 - 27)	0.07	6.2E-01	7.0E-01
**Oxidative phosphorylation**					
Phosphoric acid	4.2 (3.5 - 5.4)	2.6 (2.1 - 3.4)	0.65	4.6E-09	3.8E-08
**Lipid metabolism**					
**Glycerolipid metabolism**					
Choline	19 (16 - 25)	18 (16 - 21)	0.06	5.3E-01	6.4E-01
Ethanolamine	8.6E-2 (6.9E-2 - 1.1E-1)	1.0E-1 (8.7E-2 - 1.3E-1)	-0.33	7.6E-05	2.8E-04
Glycerol	8.0E-1 (5.5E-1 - 1.2)	9.0E-1 (6.8E-1 - 1.4)	-0.24	5.7E-02	1.0E-01
Glycerol-1-phosphate	2.7E-2 (2.0E-2 - 3.5E-2)	3.4E-2 (2.7E-2 - 4.3E-2)	-0.39	7.2E-04	2.2E-03

**Lipids**					
Dodecanoic acid	6.3E-2 (4.8E-2 - 8.0E-2)	6.1E-2 (4.8E-2 - 7.3E-2)	0.01	5.7E-01	6.6E-01
Linoleic acid	3.4E-1 (1.9E-1 - 6.1E-1)	2.9E-1 (1.6E-1 - 4.4E-1)	0.46	7.5E-02	1.3E-01
Oleic acid	3.1 (1.8 - 4.7)	2.0 (1.1 - 4.1)	0.33	3.8E-02	7.3E-02
Tetradecanoic acid	2.4E-1 (1.9E-1 - 3.1E-1)	2.2E-1 (1.7E-1 - 2.7E-1)	0.14	7.5E-02	1.3E-01
**Primary bile acid biosynthesis**					
Taurine	2.7 (1.7 - 4.4)	3.3 (2.5 - 4.9)	-0.22	6.1E-02	1.1E-01
**Metabolism of cofactors**					
**Cofactor biosynthesis**					
alpha-tocopherol	1.4E-2 (7.4E-3 - 2.4E-2)	1.1E-2 (5.9E-3 - 2.0E-2)	0.24	2.0E-01	2.9E-01
**Nucleotide metabolism**					
**Purine & pirymidine**					
Ethylmalonic acid	7.5E-1 (5.2E-1 - 9.7E-1)	7.7E-1 (6.3E-1 - 1.1)	-0.12	8.4E-02	1.4E-01
Hypoxanthine	4.9E-2 (3.1E-2 - 8.4E-2)	1.2E-1 (4.6E-2 - 2.7E-1)	-1.41	7.9E-06	3.5E-05
Ribonic acid	1.3E-2 (7.6E-3 - 2.1E-2)	1.4E-2 (1.1E-2 - 2.4E-2)	-0.22	7.7E-02	1.3E-01
Uracil	2.2E-3 (1.7E-3 - 2.8E-3)	1.7E-3 (1.2E-3 - 2.0E-3)	0.51	5.7E-05	2.2E-04
Uric acid	4.7 (2.8 - 7.6)	7.5 (5.2 - 9.6)	-0.48	1.1E-04	4.0E-04
**Xenobiotic metabolism**					
**Benzoate degradation**					
4-Hydroxybenzoic acid	1.7E-2 (1.2E-2 - 2.1E-2)	2.2E-2 (1.8E-2 - 2.7E-2)	-0.65	2.3E-06	1.2E-05

Results are shown as the molar percentage of total metabolites. Median ± interquartile range (25 – 75) was calculated, and statistical significance was assessed with Wilcoxon rank-sum test and false-discovery rate (FDR q<0.05) correction by the Benjamini-Hochberg method. SAH, s-adenosylhomocisteine; SAM, s-adenosylmethionine; THF, tetrahydrofolate; TMAO, trymethylamine N-oxide.

Supplementary Table S6. Urine metabolites from COVID-19 positive and negative patients

Metabolites (ISR/mmol creatinine percentage)	Covid-19 (n = 126)	No-Covid-19 (n = 45)	log_2FC (Covid-19 vs No-Covid-19)	p-value	q-value
**Amino acid metabolism**					
**Alanine & aspartate metabolism**					
2-ketoisocaproic acid	9.7E-3 (7.2E-3 - 1.4E-2)	9.4E-3 (7.0E-3 - 1.3E-2)	0.11	6.7E-01	7.5E-01
Alanine	1.8 (1.2 - 2.7)	2.2 (1.4 - 2.9)	-0.16	2.0E-01	3.0E-01
Aspartic acid	8.1E-2 (6.5E-2 - 1.1E-1)	7.9E-2 (6.0E-2 - 1.3E-1)	-0.09	1	1
Glutamic acid	3.9E-1 (2.3E-1 - 5.6E-1)	3.3E-1 (2.0E-1 - 5.1E-1)	0.29	2.6E-01	3.5E-01
N-methylglutamic acid	3.9E-3 (1.7E-3 - 8.6E-3)	2.5E-3 (1.1E-3 - 7.7E-3)	0.27	1.3E-01	2.1E-01
**Arginine & proline metabolism**					
Oxoproline	4.3E-1 (3.1E-1 - 5.7E-1)	4.3E-1 (2.9E-1 - 6.3E-1)	-0.03	7.2E-01	7.9E-01
**Cysteine & methionine metabolism**					
Methionine	4.5 (3.6 - 5.9)	4.6 (3.7 - 5.9)	-0.05	8.7E-01	9.0E-01
**Glycine & serine metabolism**					
Glyceric acid	3.9E-1 (2.5E-1 - 5.9E-1)	3.4E-1 (2.1E-1 - 5.6E-1)	0.24	1.7E-01	2.5E-01
Glycine	3.1 (2.5 - 3.8)	2.7 (2.1 - 3.5)	0.21	3.5E-02	6.9E-02
Serine	2.0 (1.2 - 3.0)	1.5 (8.1E-1 - 2.3)	0.46	8.2E-03	1.9E-02
Threonine	8.1E-1 (5.2E-1 - 1.3)	6.6E-1 (2.7E-1 - 8.7E-1)	0.49	9.8E-03	2.2E-02
**Histidine metabolism**					
5-hydroxyindole-3-acetic acid	9.7E-2 (6.1E-2 - 1.6E-1)	1.2E-1 (8.0E-2 - 1.7E-1)	-0.65	4.6E-02	8.6E-02
**Phenylalanine metabolism**					
4-hydroxyPhenylacetic acid	4.6E-1 (2.4E-1 - 1.6E+0)	4.3E-1 (2.9E-1 - 7.1E-1)	0.54	6.1E-01	7.0E-01
Benzoic acid	7.3E-2 (4.4E-2 - 1.2E-1)	9.8E-2 (5.4E-2 - 2.2E-1)	-1.50	1.9E-02	4.0E-02
Hippuric acid	1.7 (6.2E-1 - 4.8)	3.4 (1.8 - 5.9)	-0.53	4.1E-03	1.0E-02
Phenylalanine	6.6E-1 (4.0E-1 - 9.4E-1)	5.0E-1 (3.2E-1 - 6.7E-1)	0.42	1.1E-02	2.5E-02
**Protein digestion and absorption**					
p-Cresol	8.6E-1 (2.5E-1 - 2.1)	1.3 (7.0E-1 - 2.6)	-0.47	4.6E-02	8.6E-02
**Tryptophan metabolism**					
5-hydroxyindole-3-acetic acid	9.7E-2 (6.1E-2 - 1.6E-1)	1.2E-1 (8.0E-2 - 1.7E-1)	-0.17	1.5E-01	2.4E-01
Quinolinic acid	6.6E-2 (3.7E-2 - 1.5E-1)	6.4E-2 (2.9E-2 - 9.0E-2)	1.12	1.4E-01	2.2E-01
**Tyrosine metabolism**					
Homovanillic	8.8E-2 (5.9E-2 - 1.2E-1)	8.3E-2 (5.9E-2 - 1.1E-1)	-0.18	5.7E-01	6.6E-01
3,4-Dihydroxyphenylacetic acid	7.6E-2 (3.8E-2 - 1.6E-1)	1.5E-1 (1.0E-1 - 2.3E-1)	-0.97	8.3E-04	2.4E-03
Hydroxyphenyllactic acid	8.7E-2 (3.9E-2 - 1.7E-1)	7.3E-2 (2.4E-2 - 1.5E-1)	-0.05	4.3E-02	8.2E-02
Vanillylmandelic acid	1.7E-1 (1.1E-1 - 2.7E-1)	1.8E-1 (1.1E-1 - 3.4E-1)	-0.12	4.7E-01	5.8E-01
4-hydroxyPhenylpyruvic acid	3.1E-2 (2.1E-2 - 4.4E-2)	2.7E-2 (1.9E-2 - 4.3E-2)	0.23	1.9E-01	2.9E-01
Tyrosine	6.4E-1 (2.7E-1 - 2.8)	5.0E-1 (1.7E-1 - 1.6)	0.74	1.9E-01	2.8E-01
**Valine, leucine & isoleucine metabolism**					
2-HydroxyButyric acid	1.9E-1 (1.2E-1 - 3.6E-1)	1.8E-1 (1.2E-1 - 2.9E-1)	0.09	4.0E-01	5.1E-01
2-Hydroxyisobutyric acid	1.6 (1.0 - 2.6)	1.8 (9.6E-1 - 2.2)	0	8.1E-01	8.6E-01
2-Hydroxyisovaleric acid	1.2E-1 (6.3E-2 - 2.6E-1)	1.0E-1 (5.7E-2 - 2.0E-1)	0.53	6.7E-03	1.6E-02
2-keto-3-methylvaleric acid	1.6E-2 (1.1E-2 - 2.6E-2)	1.3E-2 (8.2E-3 - 2.3E-2)	0.11	2.0E-01	2.9E-01
3-hydroxy-2-Methylbutanoic acid	2.0E-1 (1.3E-1 - 3.0E-1)	1.6E-1 (1.1E-1 - 2.8E-1)	0.21	1.1E-01	1.8E-01
3-hydroxy-3-Methylglutaric acid	3.6E-2 (2.5E-2 - 5.6E-2)	4.2E-2 (3.3E-2 - 6.2E-2)	-0.27	7.8E-02	1.3E-01
3-hydroxybutyric acid/3-hydroxyisobutyric acid	1.1E-1 (6.8E-2 - 2.3E-1)	1.2E-1 (5.8E-2 - 1.9E-1)	1.10	7.8E-01	8.3E-01
3-Hydroxyisovaleric acid	1.4 (7.6E-1 - 2.3)	9.6E-1 (4.4E-1 - 1.5)	0.53	6.7E-03	1.6E-02

3-Methylglutaconic acid	7.2E-2 (4.7E-2 - 1.1E-1)	8.0E-2 (4.8E-2 - 1.4E-1)	-0.66	4.3E-01	5.4E-01
3-Methylglutaric acid	4.8E-2 (2.9E-2 - 7.0E-2)	4.2E-2 (2.8E-2 - 6.0E-2)	0.35	2.9E-01	3.9E-01
Isoleucine	1.1E-1 (8.2E-2 - 1.5E-1)	7.7E-2 (3.9E-2 - 1.1E-1)	0.53	4.8E-04	1.5E-03
Leucine	3.0E-1 (2.1E-1 - 4.3E-1)	2.2E-1 (1.1E-1 - 2.8E-1)	0.61	4.7E-04	1.5E-03
Methylmalonic	1.3E-2 (7.0E-3 - 2.2E-2)	2.1E-2 (8.7E-3 - 3.1E-2)	-0.30	6.1E-02	1.1E-01
Tiglylglycine	6.9E-2 (4.1E-2 - 1.1E-1)	6.1E-2 (3.9E-2 - 1.1E-1)	0.02	6.2E-01	7.0E-01
Valine	3.9E-1 (2.7E-1 - 5.5E-1)	2.7E-1 (1.5E-1 - 4.1E-1)	0.65	4.0E-04	1.3E-03
**Biosynthesis of secondary metabolites**					
**Biosynthesis of various alkaloids**					
3-hydroxypropanoic acid	2.4E-1 (1.4E-1 - 4.2E-1)	1.9E-1 (1.0E-1 - 4.2E-1)	-0.11	4.2E-01	5.3E-01
**Flavanoid metabolism**					
Quinic acid	1.7E-1 (6.2E-2 - 3.7E-1)	4.7E-1 (1.1E-1 - 1.2)	-1.07	1.3E-04	4.3E-04
**Carbohydrate metabolism**					
**Ascorbate and aldrate metabolism**					
Saccharic acid	1.6E-1 (9.7E-2 - 2.6E-1)	1.1E-1 (5.5E-2 - 1.6E-1)	0.98	1.4E-03	3.8E-03
**C5-Branched dibasic acid metabolism**					
Methylsuccinic	5.8E-2 (4.1E-2 - 9.4E-2)	6.3E-2 (5.2E-2 - 1.2E-1)	-0.01	1.7E-01	2.5E-01
**Fructose & mannose metabolism**					
1-Deoxysorbitol	1.2 (8.0E-1 - 1.9)	1.7 (1.1 - 2.2)	-0.29	4.9E-02	9.0E-02
Allose	2.8E-1 (1.6E-1 - 4.0E-1)	2.9E-1 (1.6E-1 - 4.2E-1)	-0.12	7.6E-01	8.2E-01
d-Fructose	3.5E-2 (2.4E-2 - 6.2E-2)	4.0E-2 (2.5E-2 - 7.3E-2)	0.12	5.5E-01	6.5E-01
d-Mannitol	5.5E-1 (2.6E-1 - 1.7)	4.3E-1 (2.1E-1 - 1.1)	0.53	1.4E-01	2.2E-01
d-Psicose	4.4E-2 (2.6E-2 - 8.9E-2)	4.3E-2 (2.8E-2 - 7.6E-2)	0.39	8.7E-01	9.0E-01
d-Sorbitol	8.6E-2 (4.7E-2 - 1.3E-1)	7.9E-2 (3.9E-2 - 1.2E-1)	-0.74	3.5E-01	4.6E-01
l-Fucose	4.6E-2 (3.0E-2 - 6.1E-2)	4.3E-2 (2.5E-2 - 6.8E-2)	-0.09	9.3E-01	9.5E-01
**Galactose metabolism**					
d-Galactitol	4.3E-2 (3.2E-2 - 5.5E-2)	5.0E-2 (3.2E-2 - 6.5E-2)	-0.17	1.6E-01	2.4E-01
d-Galactose	3.6 (2.7 - 5.0)	3.2 (2.3 - 3.8)	0.05	2.8E-01	3.8E-01
d-Galactose	1.0E-1 (8.5E-2 - 1.4E-1)	9.8E-2 (7.5E-2 - 1.2E-1)	0.23	4.3E-02	8.2E-02
**Glycolisis**					
Citramalic acid	6.1E-3 (3.9E-3 - 9.3E-3)	1.0E-2 (7.1E-3 - 1.6E-2)	-0.62	1.0E-04	3.7E-04
d-Glucose	4.6E-1 (3.5E-1 - 6.5E-1)	3.7E-1 (1.7E-1 - 5.2E-1)	-0.09	3.2E-02	6.4E-02
d-Glucose	8.4E-2 (6.5E-2 - 1.2E-1)	6.7E-2 (4.2E-2 - 1.1E-1)	0.33	1.1E-02	2.5E-02
Lactic acid	1.1 (7.9E-1 - 2.1)	1.7 (1.1 - 3.7)	-0.94	9.7E-04	2.8E-03
**Glyxolate and decarboxylate metabolism**					
Glycolic acid	7.7E-1 (5.2E-1 - 1.4)	7.0E-1 (3.7E-1 - 1.3)	0.17	4.0E-01	5.1E-01
**Nucleotide sugar metabolism**					
d-Arabinose	2.3E-2 (1.4E-2 - 4.0E-2)	2.2E-2 (1.4E-2 - 3.7E-2)	0.52	5.6E-01	6.6E-01
d-Glucuronic acid	1.1E-1 (6.4E-2 - 3.7E-1)	6.1E-2 (4.0E-2 - 1.1E-1)	1.75	3.7E-05	1.5E-04
d-Threitol	2.3E-1 (1.8E-1 - 2.8E-1)	2.6E-1 (1.9E-1 - 3.1E-1)	-0.19	2.4E-02	4.9E-02
d-Xylose	4.9E-3 (3.1E-3 - 8.3E-3)	4.4E-3 (2.7E-3 - 6.8E-3)	0.60	1.4E-01	2.2E-01
Erythronic acid	7.8 (5.9 - 11)	8.1 (6.2 - 10)	0.15	8.5E-01	8.9E-01
Threonic acid	7.7E-1 (4.9E-1 - 1.1)	7.3E-1 (4.7E-1 - 1.2)	-0.36	8.3E-01	8.8E-01
Xylonic acid	4.4E-1 (2.9E-1 - 6.7E-1)	5.0E-1 (3.3E-1 - 7.8E-1)	-0.68	1.8E-01	2.7E-01
**Pentose glucoronate interconversion metabolism**					
d-Arabitol	1.2E-1 (9.8E-2 - 1.6E-1)	1.4E-1 (1.0E-1 - 1.7E-1)	-0.08	3.9E-01	5.0E-01
d-Gluconic acid	8.3E-2 (5.1E-2 - 1.6E-1)	6.1E-2 (4.3E-2 - 1.0E-1)	0.67	3.6E-02	7.2E-02
d-Ribose	1.7E-2 (1.1E-2 - 2.3E-2)	1.4E-2 (9.1E-3 - 1.8E-2)	0.40	4.1E-02	7.9E-02
d-Xylitol	2.6E-2 (1.5E-2 - 5.8E-2)	1.6E-2 (1.1E-2 - 2.3E-2)	1.85	1.8E-04	6.2E-04
Galacturonic acid	1.2E-1 (5.8E-2 - 2.9E-1)	5.9E-2 (3.1E-2 - 9.3E-2)	1.74	1.9E-05	8.0E-05
meso-Erythritol	2.6 (1.6 - 3.9)	2.8 (2.0 - 5.5)	-0.11	3.3E-01	4.3E-01
myo-Inositol	4.9E-2 (2.2E-2 - 1.1E-1)	2.6E-2 (1.2E-2 - 5.6E-2)	0.95	2.3E-03	6.1E-03
Xylulose	1.9E-1 (1.3E-1 - 3.6E-1)	1.6E-1 (9.7E-2 - 2.2E-1)	1.04	9.1E-03	2.1E-02
**Sucrose metabolism**					
d-Lactose	6.3E-1 (4.3E-1 - 8.9E-1)	6.5E-1 (4.2E-1 - 8.7E-1)	0.07	7.5E-01	8.1E-01
d-Lactose	6.9E-1 (4.7E-1 - 9.9E-1)	6.7E-1 (4.3E-1 - 9.5E-1)	0.09	7.0E-01	7.7E-01
d-Maltose	8.5E-2 (2.9E-2 - 2.8E-1)	1.1E-1 (3.4E-2 - 3.9E-1)	-0.24	3.0E-01	4.0E-01
d-Sucrose	2.6E-1 (1.2E-1 - 4.6E-1)	3.0E-1 (1.2E-1 - 6.4E-1)	-0.61	3.1E-01	4.1E-01
**Tricarboxylic acid cycle metabolism**					

2-Hydroxyglutaric acid	5.9E-2 (3.1E-2 - 1.0E-1)	6.3E-2 (3.8E-2 - 1.1E-1)	-0.16	3.3E-01	4.3E-01
2-methylcitric acid	7.2E-2 (5.4E-2 - 1.1E-1)	8.0E-2 (5.2E-2 - 1.3E-1)	-0.54	5.5E-01	6.5E-01
Aconitic acid	3.5E-1 (2.1E-1 - 5.9E-1)	4.5E-1 (2.5E-1 - 5.9E-1)	-0.18	1.8E-01	2.7E-01
alpha-ketoglutaric acid	5.0E-1 (3.8E-1 - 8.5E-1)	5.5E-1 (3.5E-1 - 7.9E-1)	-0.23	9.6E-01	9.8E-01
Citric acid	2.2 (9.8E-1 - 4.4)	3.1 (9.2E-1 - 5.5)	-0.65	3.4E-01	4.4E-01
Fumaric acid	2.3E-1 (1.6E-1 - 3.7E-1)	2.1E-1 (1.3E-1 - 3.4E-1)	0.03	2.9E-01	3.9E-01
Glutamine	9.8E-3 (1.9E-3 - 4.3E-2)	6.3E-3 (1.9E-3 - 4.5E-2)	0.45	7.1E-01	7.8E-01
Malic acid	4.3E-2 (2.7E-2 - 6.5E-2)	5.1E-2 (2.5E-2 - 6.8E-2)	-0.52	5.9E-01	6.8E-01
Malonic acid	3.7E-3 (2.9E-3 - 5.2E-3)	3.8E-3 (2.2E-3 - 5.0E-3)	0.07	4.3E-01	5.4E-01
Pyruvic acid	9.7E-1 (6.8E-1 - 1.5)	1.3 (7.6E-1 - 2.0)	-0.46	4.9E-02	8.9E-02
Succinic acid	1.2E-1 (7.3E-2 - 2.1E-1)	1.3E-1 (8.6E-2 - 2.8E-1)	-0.77	4.3E-01	5.4E-01
Tartaric acid	1.4E-2 (7.6E-3 - 7.1E-2)	1.7E-2 (7.1E-3 - 4.5E-2)	1.39	9.0E-01	9.2E-01
**Lipid metabolism**					
**Glycerolipid metabolism**					
Ethanolamine	3.4 (2.2 - 6.1)	3.1 (1.8 - 5.0)	0.18	4.2E-01	5.3E-01
Glycerol	7.0E-3 (5.2E-3 - 9.7E-3)	4.7E-3 (3.4E-3 - 6.6E-3)	0.87	1.1E-04	4.0E-04
Glycerol-1-phosphate	9.0E-3 (5.9E-3 - 1.4E-2)	8.5E-3 (5.7E-3 - 1.3E-2)	0.06	9.0E-01	9.2E-01
**Lipids**					
Palmitic acid	1.2 (9.1 - 1.9)	1.4E+1 (9.7 - 2.1E+1)	-0.06	5.3E-01	6.4E-01
Suberic acid	5.7E-2 (4.0E-2 - 8.7E-2)	6.3E-2 (4.0E-2 - 8.9E-2)	0.03	8.8E-01	9.1E-01
**Metabolism of cofactors**					
**Cofactor biosynthesis**					
4-pyridoxic acid	1.0E-2 (7.2E-3 - 1.7E-2)	9.6E-3 (6.8E-3 - 1.7E-2)	-2.44	5.1E-01	6.2E-01
**Nucleotide metabolism**					
**Purine & pirymidine**					
Adenine	2.0E-1 (1.0E-1 - 3.8E-1)	1.7E-1 (1.0E-1 - 2.5E-1)	0.27	2.2E-01	3.1E-01
Dihydroorotic acid	9.0E-1 (6.4E-1 - 1.3)	8.5E-1 (6.1E-1 - 1.1)	0.23	1.7E-01	2.6E-01
Hypoxanthine	9.5E-1 (4.0E-1 - 2.2E+0)	5.7E-1 (2.7E-1 - 1.3)	0.40	1.2E-01	2.0E-01
Orotic acid	3.5E-2 (1.9E-2 - 5.3E-2)	3.6E-2 (2.1E-2 - 7.6E-2)	-0.40	2.9E-01	3.9E-01
Oxalic acid	5.9E-1 (3.6E-1 - 8.1E-1)	4.6E-1 (3.1E-1 - 6.9E-1)	0.36	6.1E-02	1.1E-01
Ribonic acid	1.3E-1 (7.5E-2 - 2.1E-1)	1.8E-1 (1.0E-1 - 2.9E-1)	-0.27	3.1E-02	6.4E-02
Thymine	1.9E-3 (1.3E-3 - 2.7E-3)	2.2E-3 (1.4E-3 - 3.2E-3)	0.00	5.6E-01	6.6E-01
Uracil	5.3E-1 (3.0E-1 - 7.9E-1)	5.4E-1 (3.5E-1 - 8.1E-1)	-0.29	5.5E-01	6.5E-01
Uric acid	6.4 (4.6 - 8.2)	5.2 (3.4 - 7.2)	0.28	1.5E-02	3.3E-02
Xanthine	1.9E-2 (1.1E-2 - 3.0E-2)	1.6E-2 (1.1E-2 - 2.4E-2)	-0.21	5.3E-01	6.4E-01
**Xenobiotic metabolism**					
**Benzoate degradation**					
4-Hydroxybenzoic acid	1.0E-1 (6.1E-2 - 1.8E-1)	1.4E-1 (9.3E-2 - 2.4E-1)	-0.13	3.7E-02	7.2E-02
**Caprolactam degradation**					
Adipic acid	2.2E-1 (1.2E-1 - 5.2E-1)	2.0E-1 (8.8E-2 - 4.6E-1)	0.18	3.1E-01	4.1E-01

Results are shown as the internal standard/mmol creatinine percentage of total metabolite. Median ± interquartile range (25 – 75) was calculated, and statistical significance was assessed with Wilcoxon rank-sum test and false-discovery rate (FDR q<0.05) correction by the Benjamini-Hochberg method.
